# A Mutation in the *Srrm4* Gene Causes Alternative Splicing Defects and Deafness in the Bronx Waltzer Mouse

**DOI:** 10.1371/journal.pgen.1002966

**Published:** 2012-10-04

**Authors:** Yoko Nakano, Israt Jahan, Gregory Bonde, Xingshen Sun, Michael S. Hildebrand, John F. Engelhardt, Richard J. H. Smith, Robert A. Cornell, Bernd Fritzsch, Botond Bánfi

**Affiliations:** 1 Department of Anatomy and Cell Biology, Carver College of Medicine, University of Iowa, Iowa City, Iowa, United States of America; 2 Inflammation Program, Carver College of Medicine, University of Iowa, Iowa City, Iowa, United States of America; 3 Department of Biology, College of Liberal Arts and Sciences, University of Iowa, Iowa City, Iowa, United States of America; 4 Department of Otolaryngology–Head and Neck Surgery, Carver College of Medicine, University of Iowa, Iowa City, Iowa, United States of America; 5 Department of Internal Medicine, Carver College of Medicine, University of Iowa, Iowa City, Iowa, United States of America; Stanford University School of Medicine, United States of America

## Abstract

Sensory hair cells are essential for hearing and balance. Their development from epithelial precursors has been extensively characterized with respect to transcriptional regulation, but not in terms of posttranscriptional influences. Here we report on the identification and functional characterization of an alternative-splicing regulator whose inactivation is responsible for defective hair-cell development, deafness, and impaired balance in the spontaneous mutant Bronx waltzer (bv) mouse. We used positional cloning and transgenic rescue to locate the *bv* mutation to the splicing factor-encoding gene *Ser/Arg repetitive matrix 4* (*Srrm4*). Transcriptome-wide analysis of pre–mRNA splicing in the sensory patches of embryonic inner ears revealed that specific alternative exons were skipped at abnormally high rates in the bv mice. Minigene experiments in a heterologous expression system confirmed that these skipped exons require Srrm4 for inclusion into the mature mRNA. Sequence analysis and mutagenesis experiments showed that the affected transcripts share a novel motif that is necessary for the Srrm4-dependent alternative splicing. Functional annotations and protein–protein interaction data indicated that the encoded proteins cluster in the secretion and neurotransmission pathways. In addition, the splicing of a few transcriptional regulators was found to be Srrm4 dependent, and several of the genes known to be targeted by these regulators were expressed at reduced levels in the bv mice. Although Srrm4 expression was detected in neural tissues as well as hair cells, analyses of the bv mouse cerebellum and neocortex failed to detect splicing defects. Our data suggest that Srrm4 function is critical in the hearing and balance organs, but not in all neural tissues. Srrm4 is the first alternative-splicing regulator to be associated with hearing, and the analysis of bv mice provides exon-level insights into hair-cell development.

## Author Summary

The identification of novel deafness-causing mutations has been instrumental in revealing unexpected mechanisms that are required for development of the sound- and gravity-sensing hair cells of the inner ear. The Bronx waltzer (bv) mouse is characterized by defects in hair-cell development, as well as by deafness and impaired balance. Here, we report on our identification of a mutation in the *Ser/Arg repetitive matrix 4* (*Srrm4*) gene as the source of these defects. The encoded protein, Srrm4, belongs to a family of RNA splicing factors that regulate the inclusion of certain genetic information (i.e. alternative exons) into the transcribed RNA. We analyzed the molecular function of Srrm4 by comparing the exon composition of RNAs in the inner ear of bv and control mice. This approach revealed that, in the bv mice, specific alternative exons were omitted from protein-encoding RNAs. The affected transcripts shared two features: they contained a short sequence motif that was required for Srrm4-dependent splicing, and they encoded proteins that were related predominantly to secretion and neurotransmission. In addition, RNAs of a few gene expression regulators contained Srrm4-regulated exons. Our data suggest that Srrm4-dependent alternative splicing has a profound effect on the developmental program of hair cells.

## Introduction

Hair cells of the hearing and balance organs are specialized mechanoreceptors that convert mechanical stimuli to electrical signals. These signals are transmitted to the central nervous system via connecting afferent neurons. Hair cells of the hearing organ are specialized further as inner hair cells (IHCs) and outer hair cells (OHCs). IHCs are the primary auditory receptors, whereas the electromotile OHCs (and their neural feedback loops) are amplifiers of the mechanical stimulus [1]. In mice, hair cells become responsive to mechanical stimuli between embryonic day (E)17 and postnatal day (P)4 [2], [3]. During this period, the mechanosensing stereociliary bundles of the hair cells grow and become organized into rows of increasing height [4]. Defects in either stereocilium formation or afferent synaptogenesis lead to deafness and impaired balance [5]–[8].

The development of sensory hair cells is governed by several known transcription factors. For example, Sox2, Eya1, and the Notch effectors Hes1 and Hey1/2 are key transcriptional regulators of the specification process that guides the undifferentiated otocyst cells towards a prosensory fate [9]. The prosensory cells can then differentiate into either hair cells or supporting cells, depending on the presence or absence of the basic helix-loop-helix transcription factor Atoh1 within the cell. Genetic deletion of *Atoh1* leads to the complete absence of hair cells [10], whereas the ectopic expression of Atoh1 in supporting cells can induce the formation of stereociliary bundles and the expression of hair-cell markers [11]–[13]. Atoh1 also induces the expression of at least 2 other transcription factors (*i.e.* Pou4f3 and Gfi1) that are required for the terminal differentiation of hair cells [14]–[17].

Proper hair-cell differentiation has also been shown to depend on microRNA-96 (miR-96), a post-transcriptional regulator of gene expression. Mutations in the *miR-96* genes of both humans and mice have been associated with deafness [18], [19], and the analysis of mice harboring such mutations has demonstrated that this miR is required for the maturation of stereociliary bundles, as well as for the establishment of auditory nerve connections [20]. In addition, the analysis of knockout mice lacking the miR-processing protein Dicer1 in the inner ear supports the notion that miR-dependent regulation of gene expression plays a critical role in hair-cell differentiation [21], [22].

In an effort to identify additional regulatory mechanisms that are necessary for hair-cell development, we analyzed the bv mouse line, whose inner ear pathology suggested that the *bv* mutation disrupts a gene that is key to the differentiation of most hair-cell types [23]–[26]. Although the hair cells of homozygous bv (*bv/bv*) mice are morphologically intact until E17.5, neither the IHCs in the hearing organ nor the vestibular hair cells (VHCs) in the balance organs develop normally beyond this point. Specifically, the IHCs and VHCs fail to form synapses with afferent neurons, exhibit delayed stereociliary-bundle growth, and tend to degenerate by P3–5 [25]–[27]. These hair-cell defects are associated with the deafness and impaired balance observed in these animals. The *bv/bv* mouse is unique amongst the deaf mouse models in that IHC degeneration is not accompanied by the loss of OHCs [23], [27].

In this study, we localize the deafness-causing gene defect of the bv mouse line to the splicing factor-encoding gene *Srrm4* (also known as *nSR100* [28]). Because Srrm4 is expressed broadly in neural tissues, we used tissue-selective transgenic rescue to examine the biological importance of Srrm4 in and outside of the inner ear. The results of these rescue experiments indicated that defective Srrm4 function specifically in hair cells is the main, if not the only, cause of the bv phenotype. We evaluated the molecular function of Srrm4 using a transcriptome-wide approach, and found that it was required for neuron-like alternative splicing in the developing sensory patches of the inner ear but not in other Srrm4-expressing tissues that we examined. The majority of the affected pre-mRNAs encoded proteins with functions related to neurotransmission and secretion, confirming the notion that alternative splicing factors can selectively alter specific functional modules in the cell. Moreover, Srrm4-dependent splicing in hair cells affected transcriptional regulators that are known to control cell differentiation and presynaptic vesicle processing in neural tissues. Thus, our analysis of the bv mouse line suggests that Srrm4-dependent regulation of alternative exon choice has a profound effect on the differentiation program of sensory hair cells.

## Results

### The Bronx waltzer mouse line harbors a mutation in the *Srrm4* gene

**Figure 1:**
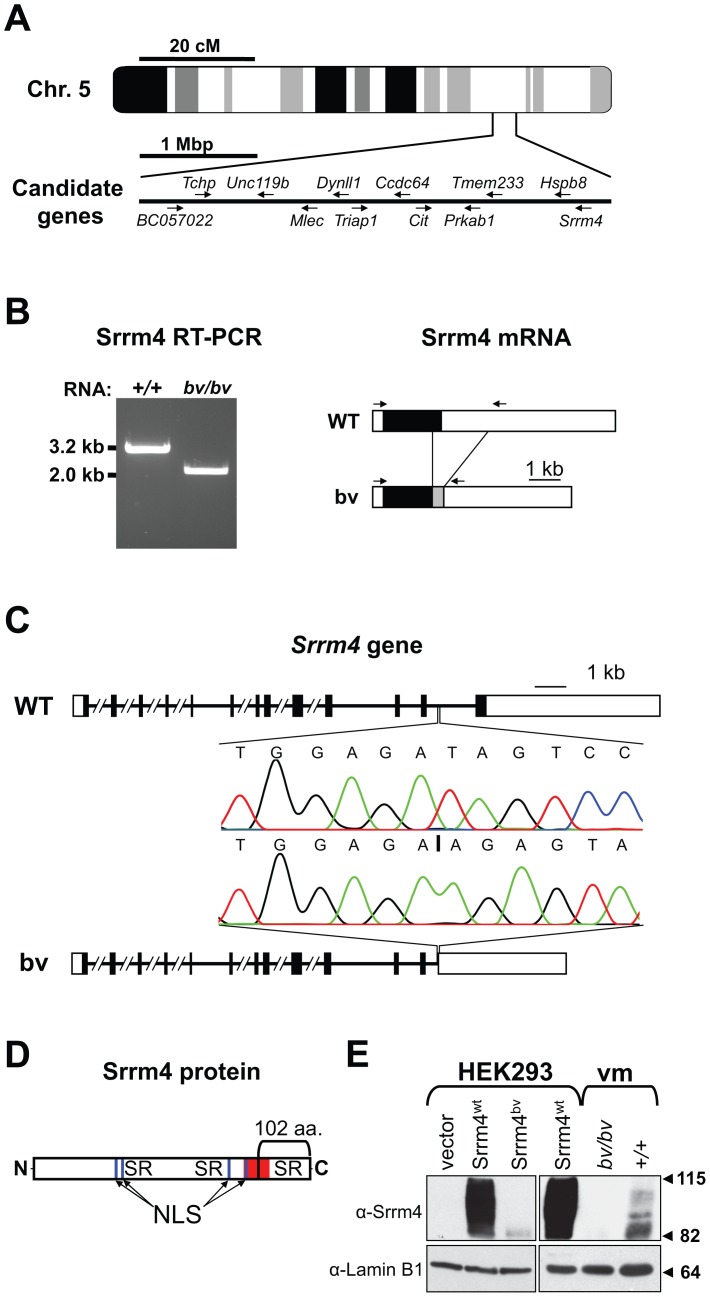
Deletion mutation in the *Srrm4* gene of bv mice. (A) Schematic representation of genomic positions of the selected candidate genes in the 4-mega base pair (Mbp) interval to which the *bv* mutation has been mapped. (B) Left: RT-PCR-based detection of an abnormally short Srrm4 transcript in the inner ear of a *bv/bv* mouse. Right: schematic representation of wild-type (WT) and bv Srrm4 transcripts showing the positions of the RT-PCR primers (arrows), the translated and non-translated regions (black and white boxes, respectively), and a normally intronic sequence in the Srrm4 mRNA of the *bv/bv* mouse (gray box). (C) Comparison of the *Srrm4* genes of wild-type and *bv/bv* mice. Horizontal lines represent introns, and black and white boxes represent the coding and non-coding regions of exons, respectively. The *Srrm4* gene of *bv/bv* mice lacks parts of the last intron and exon. Chromatograms highlight differences between the WT and *bv/bv* mice with respect to the *Srrm4* sequence near the deletion site (starting at vertical line in the lower chromatogram). (D) Schematic representation of the Srrm4 protein. The bracket indicates the portion of the protein that is encoded by the last *Srrm4* exon in wild-type mice and is lacking in the *bv/bv* mice. The Ser/Arg (SR)-rich regions, putative nuclear localization signals (NLS), and a region that is highly conserved between Srrm4 and its closest paralogue, Srrm3 (amino acids 478–525 in Srrm4), are also indicated (red). (E) Upper panels: immunoblot analysis of Srrm4 expression in transfected HEK293 cells and in the vestibular macula (vm) of *bv/bv* and *+/+* mice on E16.5. As indicated in the panel, the HEK293 cells were transfected with Srrm4^wt^, Srrm4^bv^, or an empty expression vector. Lower panels: comparable protein loading is demonstrated by the Lamin B1 signal present in all samples. Arrowheads and numbers (in kDa) indicate the positions of MW standards.

Although the gene affected by the *bv* mutation was not identified in previous studies, it had been mapped to a 4-mega base pair (bp) interval on chromosome 5 [29]. We examined the tissue expression profiles and putative functions of all 63 genes localized within this interval and selected 12 for further analysis (Figure 1A). In amplifying the transcripts of the 12 genes, we found that one, which encodes the splicing factor Srrm4, was abnormally short in *bv/bv* mice (Figure 1B). Sequence analysis showed that the shortened Srrm4 transcript lacked hundreds of nucleotides and retained an intronic sequence (Figure 1B), whereas the other amplified transcripts did not contain any mutations (data not shown). Sequencing of the 3′ end of the *Srrm4* gene in *bv/bv* mice revealed a 2,710-bp deletion that removed a portion of the last intron and the entire coding region of the last exon but left the polyadenylation site intact (Figure 1C and Table S1). The affected last exon of *Srrm4* encodes potentially important domains of the Srrm4 protein, including the C-terminal SR repeats and a region that is highly conserved between Srrm4 and its closest paralogue, Srrm3 (Figure 1D). We used Western blotting to examine expression of the wild-type Srrm4 (Srrm4^wt^) protein and that of the mutant form encoded by the bv mouse genome (Srrm4^bv^). In nuclear pellets generated from Srrm4^wt^-transfected HEK293 cells and from the sensory regions of the balance organs (*i.e.* vestibular maculas) in wild-type mice, the protein was detected as multiple bands between 82 and 115 kDa (Figure 1E). In contrast, in nuclear pellets generated from Srrm4^bv^-transfected HEK293 cells and the vestibular maculas of *bv/bv* mice, only a single, faint band was detected at ∼82 kDa (Figure 1E). Thus, the *bv* mutation not only truncates the Srrm4 protein but also interferes with either the stability or synthesis of the truncated protein.

### The Bronx waltzer phenotype is caused by defective Srrm4 function in hair cells

**Figure 2:**
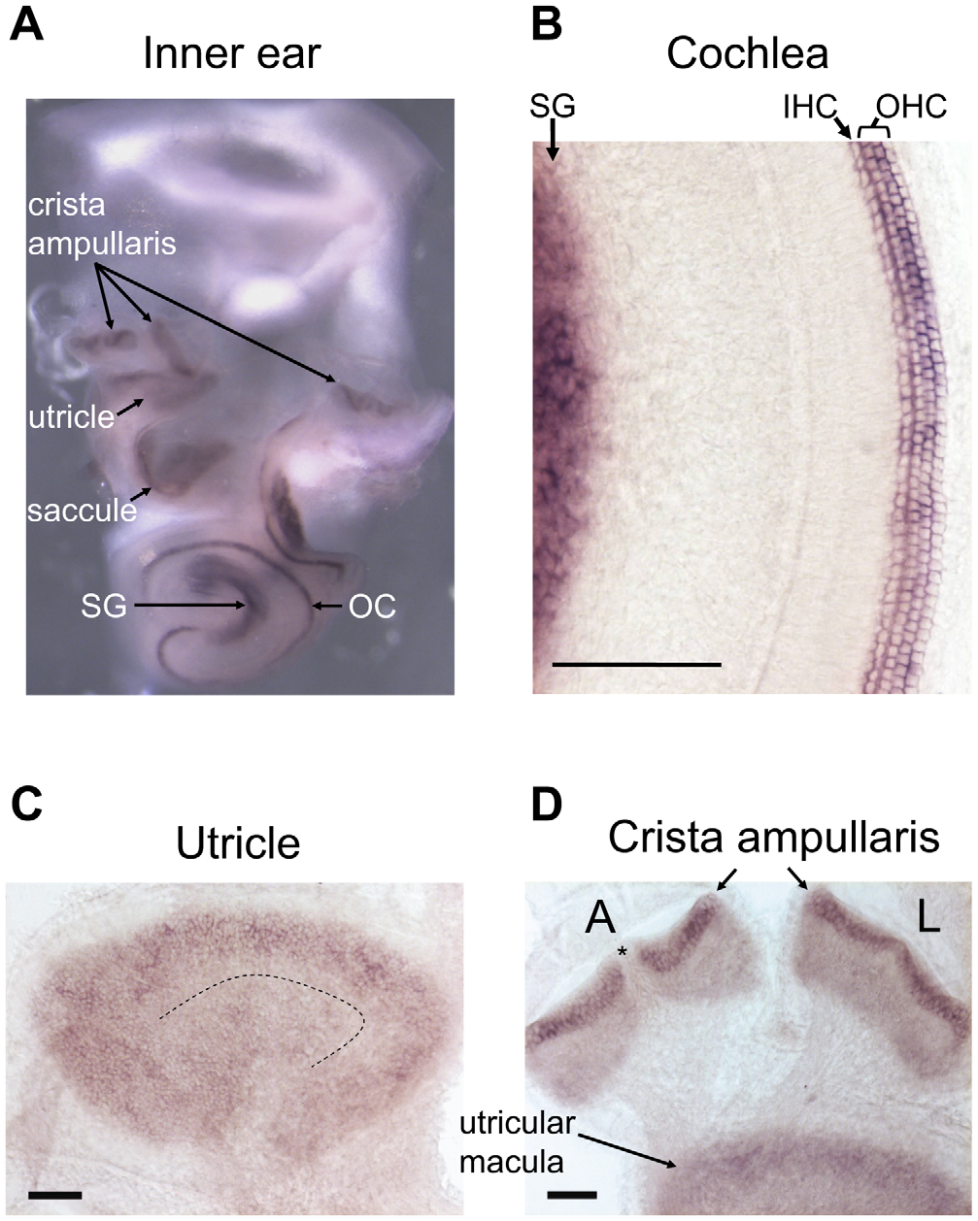
*In situ* hybridization of the mouse inner ear with an antisense Srrm4 probe. (A) Whole-mount *in situ* hybridization of the inner ear revealing Srrm4 detection in each balance organ (*i.e.* crista ampullaris, saccule, and utricle), in the organ of Corti (OC), and in the spiral ganglion (SG). (B) In the cochlea, the antisense Srrm4 probe labeled all three rows of OHCs, the row of IHCs, and the spiral ganglion (SG). (C) In the utricular macula, the Srrm4 signal was present throughout, but weaker in the central (*i.e.* striolar) region than in the periphery. The dotted line indicates the estimated center of the striola. (D) In the crista amupllaris, Srrm4 signal was present in the sensory-cell layer. The anterior (A) and lateral (L) cristae are shown. Asterisk indicates the unstained, non-sensory septum cruciatum of the anterior crista. An adjacent segment of the utricular macula is also shown. Scale bars: 100 µm.

Next, we used *in situ* hybridization to examine the expression pattern of wild-type Srrm4 in the inner ear, and found that the Srrm4 mRNA was detected in all sensory regions of hearing and balance organs (Figure 2A). In the cochlea, the antisense Srrm4 probe labeled the IHCs, OHCs, and spiral ganglion (Figure 2B). In the utricle, the most intensive staining was found at the periphery of the sensory macula (Figure 2C), where the density of VHCs is highest [30]. In the crista ampullaris, the VHC-containing regions were strongly positive, whereas the non-sensory septum cruciatum (present in the anterior but not the lateral crista [31]), was not labeled (Figure 2D, asterisk). The negative-control, Srrm4 sense probe did not hybridize with any of the sensory regions in the inner ear (Figure S1). These data indicate that Srrm4 is expressed in the sensory hair cells and the spiral ganglion. RT-PCR experiments showed that the Srrm4 mRNA was also present in the brain but not in the kidney, liver, or spleen (data not shown), consistent with the previously reported neural expression pattern of Srrm4 [28].

**Figure 3:**
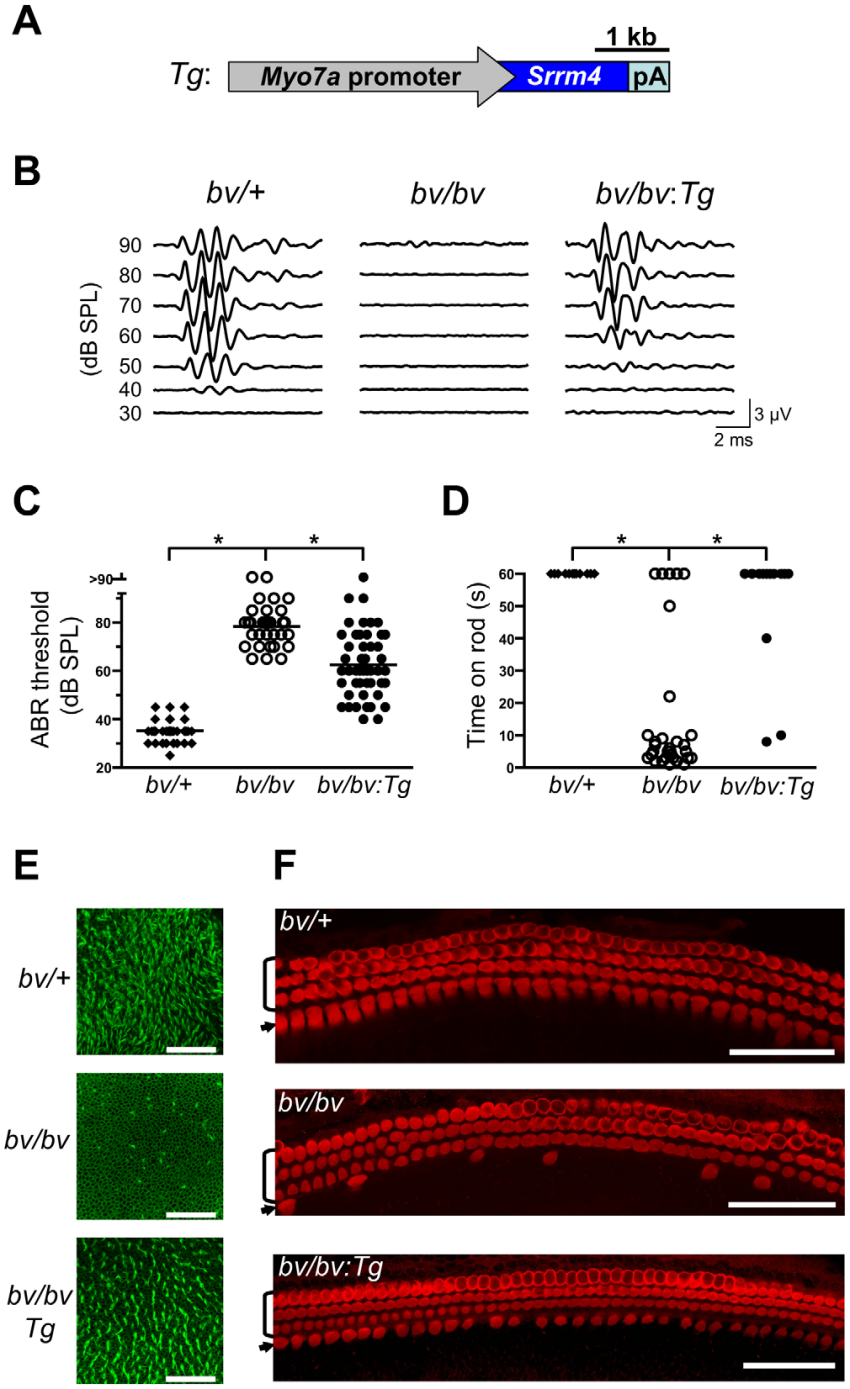
The bv phenotype is rescued by hair-cell targeted expression of an *Srrm4* transgene. (A) Schematic representation of the *Srrm4* transgene (Tg) designed for rescue experiments. It consists of a mouse *Myo7a* promoter, the Srrm4 coding sequence, and a polyadenylation (pA) site. (B) Representative ABR waveforms for *bv/+*, *bv/bv*, and *Srrm4*-transgenic *bv/bv* mice (*bv/bv:Tg*). Broadband click stimuli were applied at the indicated sound pressure levels (SPL). (C) Statistical analysis of ABR thresholds for *bv/+*, *bv/bv*, and *Srrm4*-transgenic *bv/bv* mice; each symbol represents the value for a single mouse (one-way ANOVA, *P*<0.0001, post-hoc Tukey's test: *P<0.01). (D) Time spent on a fixed horizontal rod before falling, by *bv/+*, *bv/bv*, and *Srrm4*-transgenic *bv/bv* mice at P70–80. The maximal duration of the assay was 60 s (one-way ANOVA, *P*<0.0001, post-hoc Tukey's test: *P<0.01). (E) Vestibular macula preparations from *bv/+*, *bv/bv*, and *Srrm4*-transgenic *bv/bv* mice (P5) stained with phalloidin-Alexa Fluor 488 to visualize actin-rich structures, including stereocilia. (F) Mid-turn organ of Corti preparations from *bv/+*, *bv/bv*, and *Srrm4*-transgenic *bv/bv* mice (P5) stained with an anti-Myo7a antibody, which labels specifically the IHCs (arrows) and OHCs (brackets). Only the IHC row is affected by the *bv* mutation. Scale bars: 50 µm.

Although Srrm4 is expressed broadly in neural tissues, we hypothesized that the Srrm4 defect in hair cells is the cause of the bv phenotype. We tested this possibility by transgenic rescue. Specifically, a *Myo7a-Srrm4* transgene (Figure 3A) was constructed in which the hair-cell specific promoter of *Myo7a* [32] controlled the transcription of wild-type Srrm4. Transgenic founder mice were generated via pronuclear injection of the *Myo7a-Srrm4* transgene, and bred into the *bv/bv* line. *In situ* hybridizations of inner ear samples with a probe corresponding to the coding region in *Srrm4* exon 13 demonstrated that the *Myo7a-Srrm4* transgene was expressed in the balance organs and IHCs of the transgenic *bv/bv* mice (Figure S2). In contrast, the inner ears of *bv/bv* mice did not hybridize with the Srrm4 exon 13 probe (Figure S2), confirming that exon 13 is missing from the Srrm4 transcript in the *bv/bv* mice. We evaluated the hearing of *Myo7a-Srrm4* transgenic *bv/bv* mice and non-transgenic *bv/bv* littermates by measuring the auditory brainstem response (ABR) of these animals on P21–28, using broadband sounds. The ABR measurements confirmed that the *bv/bv* mice were severely hearing impaired (Figure 3B), whereas the hearing thresholds of several *Myo7a-Srrm4* transgenic *bv/bv* mice were at the wild-type level (Figure 3B and 3C). Nevertheless, the extent to which hearing was restored in individual *Myo7a-Srrm4* transgenic *bv/bv* mice varied (Figure 3C) by transgenic lineage (Figure S3), and probably reflected differences in transgene insertion site or copy number.

We also assessed balance in the transgenic *bv/bv* animals, by measuring the length of time they could remain on a horizontal rod. The performance of the *Myo7a-Srrm4* transgenic *bv/bv* mice was similar to that of *bv/+* animals, which were able to remain on the rod for the duration of the assay (60 s); in contrast, most *bv/bv* mice fell within 10 s (Figure 3D).

We next analyzed the effects of the *Myo7a-Srrm4* transgene on hair-cell survival in *bv/bv* mice. The sensory regions were dissected from the balance organs of *bv/+*, *bv/bv*, and *Myo7a-Srrm4* transgenic *bv/bv* mice on P5, and the actin-rich stereociliary bundles of VHCs were visualized using fluorescently labeled phalloidin. Looking at the balance organs of *bv/bv* and *Myo7a-Srrm4* transgenic *bv/bv* mice, we found that the vast majority of stereociliary bundles were absent in the former but present at nearly normal density in the latter (see utricle in Figure 3E and quantitative analysis in Figure S4A). In the cochleas of *bv/bv* mice, both actin staining of stereocilia and immunofluorescence-based visualization of the hair-cell protein Myo7a indicated that 71% of the IHCs were absent on P5. In contrast, in the *Myo7a-Srrm4* transgenic *bv/bv* mice 63% of IHCs were present at this time (Figure 3F and Figure S4). These results indicate that the *Srrm4* mutation is responsible for the hair-cell loss, deafness, and balance defect in the bv mouse line. Our data also support the notion that the inner ear pathology in *bv/bv* mice is caused by defects in the hair cells rather than in the neurons.

### The Bronx waltzer mice are subject to alternative splicing defects in the inner ear but not in the cerebellum

**Figure 4:**
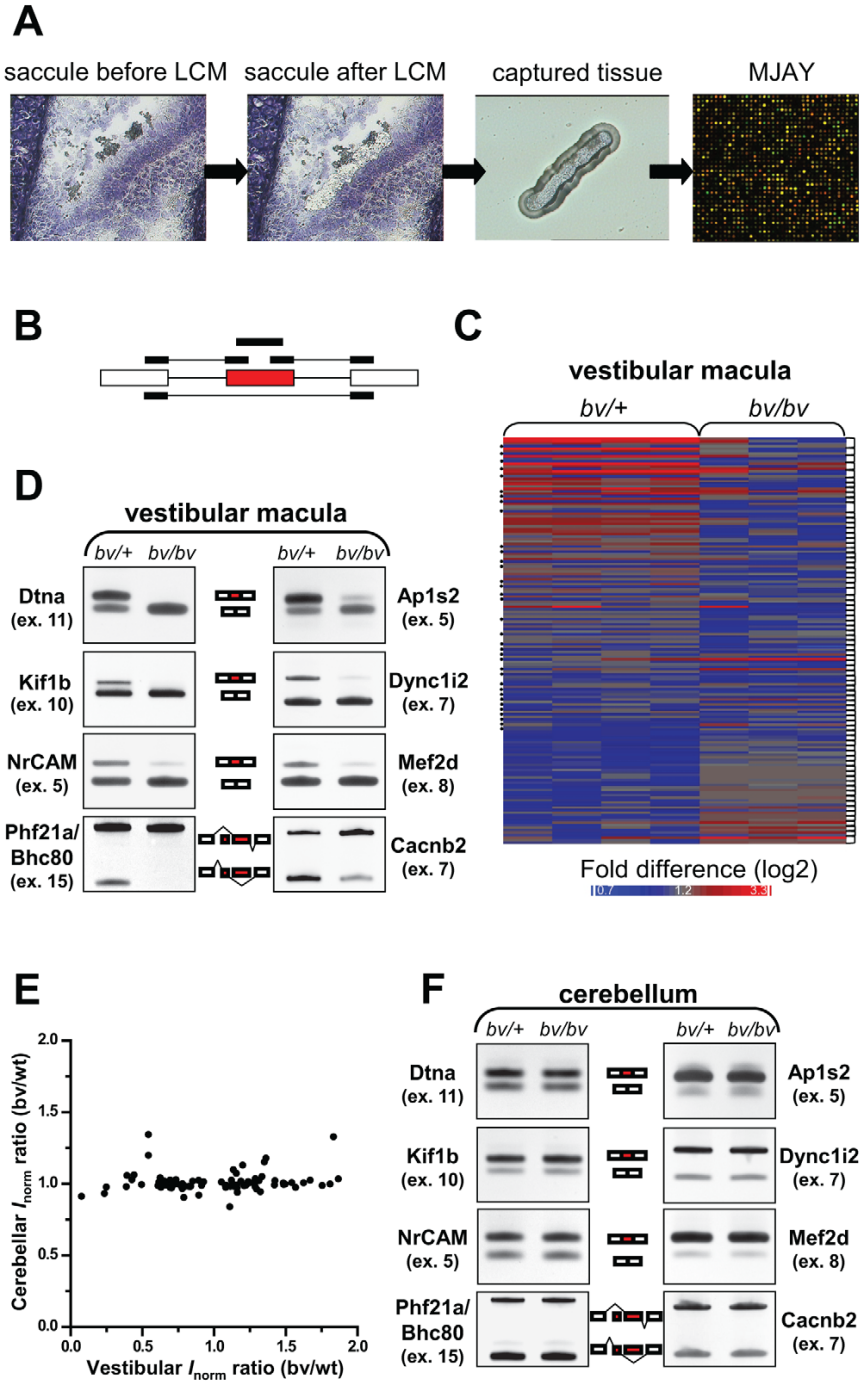
*bv/bv* mice are subject to splicing defects in the inner ear but not in the cerebellum. (A) Schematic workflow for analyzing pre-mRNA splicing in the vestibular maculas of *bv/bv* and *bv/+* mice. Vestibular maculas were isolated by laser capture microdissection (LCM), and RNA samples from the captured tissue were analyzed using mouse exon-junction microarrays (MJAYs). (B) The design of MJAY probe sets for cassette exons. Typically, 4 MJAY probe sets (black boxes) are used to measure the splicing of one cassette exon (red box); the probe sets anneal to the cassette exon itself, to the upstream and downstream exon-exon junctions, and to the skipping junction. (C) Microarray heat map of normalized probe-set signals. Probe-set signals are shown where at least two probe sets per alternative exon (connected by brackets) led to significantly different assessments of splicing rates in the vestibular maculas of *bv/+* and *bv/bv* mice. Dots at the left margin represent the data generated by exon-skipping probe sets. (D) RT-PCR validation of 8 splicing differences between the vestibular maculas of *bv/+* and *bv/bv* mice (additional RT-PCR data are shown in Figure S5). The RT-PCR primers were designed to anneal to constitutive exons (white boxes) flanking the tested alternative exons (red boxes). (E) The ratios of normalized probe-set intensities (*I*_norm_) from the vestibular maculas of *bv/+* and *bv/bv* mice are plotted against those from the cerebellums of *bv/+* and *bv/bv* mice. Only the probe sets that are indicative of splicing differences in the vestibular macula are included in the plot (Pearson's correlation test, *r* = 0.1, *P* = 0.3). (F) RT-PCR confirmation that the cerebellum of *bv/bv* mice lacks the splicing defects observed in the vestibular macula of the same mouse line.

Srrm4 belongs to the family of SR-related proteins, which act as regulators of alternative pre-mRNA splicing [33], [34]. Therefore, we examined whether alternative splicing was altered in the embryonic hair cells of *bv/bv* mice, using a transcriptome-wide approach. Specifically, embryonic hair cells (and the adjacent supporting cells) were acquired from the vestibular maculas of *bv/bv* and *bv/+* mice, by laser-capture microdissection, on E16.5, *i.e.* ∼1 day before the onset of hair-cell degeneration. RNA from the captured tissue was analyzed using the new Affymatrix chip ‘Mouse Exon Junction Array’ (MJAY, Figure 4A). MJAY contains more than half a million exon and exon-exon junction probe sets (see probe-set design in Figure 4B), and interrogates all of the splicing events supported by mouse EST/mRNA evidence within the UCSC/Ensembl databases. Processing of the MJAY data was carried out largely in the Partek Genomics Suite (see details in Methods), based on concepts that were previously described for the analysis of Human Exon Junction Array data [35]. The frequency of an alternative splicing event was considered to differ significantly between the *bv/bv* and *bv/+* samples if the difference in normalized intensities for at least two probe sets per splicing event (Figure 4C) resulted in *P*-values less than 0.05. Seventy-six candidate alternative splicing events were found and tested further by RT-PCR, using primers that annealed with the constitutive exons upstream and downstream of the alternative exons. These reactions validated 24 alternative splicing events in the vestibular maculas of *bv/bv* mice (Figure 4D and Figure S5A). Notably, examination of these splicing defects indicated that, in the *bv/bv* cells, certain alternative exons were either spliced into the mature mRNA at reduced frequency or completely skipped. Common features of the affected exons included conservation among vertebrates (data not shown) and – with the exception of Add1 exon 15 – a neuron-specific inclusion pattern (Figure S5C). Therefore, we used ‘conservation’ and ‘neuron-specific splicing’ (based on EST evidence) as new criteria with which to scrutinize the list of exons for which a single probe set suggested abnormal splicing in the *bv/bv* mice. RT-PCR revealed that, among the 283 new candidate exons, 30 were incorrectly spliced in the *bv/bv* sample (Figure S5B). Thus, overall, RT-PCR verified 54 changes in splicing in the *bv/bv* mouse (see Table S2). We used the DAVID software [36] to analyze the gene ontology (GO) annotations of the encoded proteins, and found that the lowest P values were for those associated with the ‘transmission of nerve impulse’ (Benjamini-Hochberg corrected *P*-value = 0.00047), ‘secretion by cell’ (*P*-value = 0.0064), and other closely related GO terms (*e.g.* ‘cell-cell signaling’).

The majority of splicing defects we found in the vestibular macula of *bv/bv* mice (81%) had not been reported in an earlier study that examined Srrm4 function in the Neuro2A cell line [28]. Conversely, although nPTB (exon 10) was found to be a key target of Srrm4 in the Neuro2A cells [28], it was spliced normally in the vestibular maculas of *bv/bv* mice (Figure S5D). Nevertheless, there were striking instances of overlap as well. For example, the RE1 silencing transcription factor (Rest, exon 4) was reported as a target of Srrm4 in the Neuro2A cells [37], and RT-PCR showed that the same Rest exon was differently spliced in the inner ears of *bv/bv* and *bv/+* mice (Figure S5E). Furthermore, the Srrm4-dependent splicing of Rest had been shown to affect the expression of numerous Rest-regulated genes in Neuro2A cells [37], and our analysis of the MJAY data suggested that Rest-regulated genes [38] were overrepresented among those whose expression was reduced in the vestibular macula of *bv/bv* mice (χ^2^ test *P*<0.0001, Figure S6). Notably, our data showed that the Phf21a/Bhc80 mRNA, which encodes a negative modulator of Rest-dependent transcriptional regulation [39]–[41], was also differentially spliced in the vestibular maculas of *bv/bv* and *bv/+* mice (Figure 4D). These results support the notion that Srrm4 modifies gene expression in hair cells, probably through the alternative splicing of specific transcriptional regulators.

We also wanted to test whether the *bv* mutation led to splicing alterations in Srrm4-expressing tissues other than the inner ear. We focused on the cerebellum, based on our RT-PCR analysis showing that the Srrm4 transcript is highly expressed in this tissue (Figure S7A), and *in situ* hybridization data in the Allen Brain Atlas [42] indicating that the neuron-rich layers of the cerebellum contain large amounts of Srrm4 mRNA. Notably, although the analysis of MJAY data identified 18 alternative exons as potentially differently spliced in the cerebellums of *bv/bv* and *bv/+* mice on P15, anlaysis by RT-PCR failed to validate such an outcome (Figure S7C and S7D). Furthermore, both the MJAY and RT-PCR data showed that, in the bv mouse line, inclusion rates for alternative exons that were abnormally spliced in the vestibular macula were unaltered in the cerebellum (Figure 4E and 4F and Figure S7E). Given that the Srrm4 mRNA is highly expressed in the neocortex [28], we used RT-PCR to test the inclusion rates of 10 Srrm4-regulated exons in this tissue. Again, we found no alterations in the inclusion rates of tested alternative exons in the investigated brain region of *bv/bv* mice (Figure S8). These findings are supported by the lack of obvious histological alterations in the cerebellum and neocortex of *bv/bv* mice (Figure S7B and Figure S9). In sum, the *bv* mutation does not lead to apparent defects in these Srrm4-expressing tissues.

### Srrm4-dependent alternative splicing requires the C-terminal region of Srrm4 and a novel sequence motif in the target pre–mRNA

**Figure 5:**
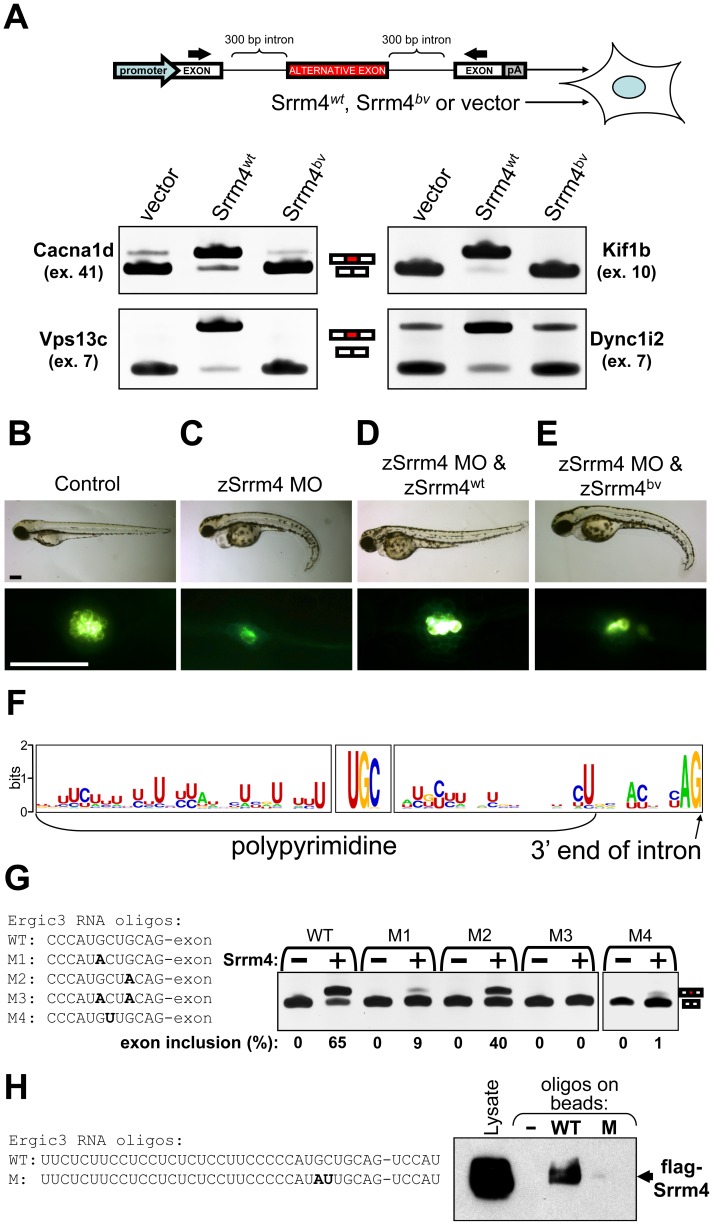
Srrm4-dependent splicing requires the C-terminal region of Srrm4 and a novel motif in the pre–mRNA. (A) RT-PCR-based testing of alternative splicing in HEK293 cells co-transfected with an Srrm4-encoding construct (Srrm4^wt^, Srrm4^bv^, or an empty expression vector) along with a minigene consisting of exons and introns (diagram). Each minigene contained an alternative exon (red box) and the adjacent intronic sequences, and these were situated between two constitutive exons (white boxes). The promoter and polyadenylation site (pA) of the minigene are indicated. Arrows indicate positions of the RT-PCR primers. Results obtained from the RT-PCR of 4 minigene-encoded mRNAs are shown. (B–E) Hair-cell survival in zebrafish injected with various combinations of the zSrrm4 MO, MO-insensitive zSrrm4^wt^ mRNA, and MO-insensitive zSrrm4^bv^ mRNA, as indicated. The upper panels show representative brightfield images of zebrafish (72 hpf) from each treatment group. The lower panels show representative fluorescence images of neuromasts from each treatment group following visualization of the hair cells with the FM1–43 dye (strong green signal). The faint green signal at cell-cell junctions is due to transgenic expression of membrane-anchored GFP in neuromasts of the zebrafish line. Scale bars: 200 µm. (F) A sequence-logo representation of the consensus sequence motifs found directly upstream of Srrm4-regulated exons. The detected consensus motifs include a polypyrimidine tract, a UGC motif, and an AG motif. (G) Results for RT-PCR testing of alternative splicing in HEK293 cells transfected with either the wild-type (WT) or a mutant (M1–4) version of an Ergic3 minigene, plus Srrm4^wt^ (+) or an empty expression vector (−), as indicated. The WT and mutated sequences (M1–4) are shown. Mutated bases are shown in bold font. (H) RNA pull down of flag-tagged Srrm4 from the whole cell lysate (Lysate) of transfected HEK293 cells, using biotinylated RNA oligos or control empty streptavidin beads (−). The sequence of the wild-type RNA oligo (WT) contained 35 nucleotides from the intron preceding the Srrm4-regulated exon and 5 nucleotides from the exon. The boundary between the intron and exon is indicated by a hyphen. In the mutated RNA oligo (M), the GC motif was substituted with AU (bold characters). The amount of flag-Srrm4 in the cell lysate and on the washed beads was evaluated using an anti-flag antibody and Western blotting.

We next used a reconstituted system to evaluate whether the Srrm4^bv^ protein retains molecular function. Specifically, HEK293 cells were transfected with Srrm4^bv^, Srrm4^wt^, or empty vector (control) alongside various minigenes consisting of exons and introns. Each minigene construct contained an exon that was incorrectly spliced in the vestibular macula of *bv/bv* mice, the flanking intronic sequences (∼300 bp), and two constitutive exons (Figure 5A). Of the 54 alternative exons whose inclusion rates were found to be altered in the vestibular maculas of *bv/bv* mice, 12 were randomly selected for these minigene experiments. RT-PCR-based evaluation of pre-mRNA splicing demonstrated that all 12 exons required Srrm4^wt^ for alternative splicing in the transfected cells, and that Srrm4^bv^ was unable to promote such splicing (Figure 5A and Figure S10A). When the minigenes were co-transfected with a construct encoding an SR protein other than Srrm4 (*i.e.* Srsf1), the inclusion rates of the alternative exons did not increase above background levels (Figure S10A). Thus, the minigene experiments confirmed that splicing of the tested exons is dependent on Srrm4^wt^, and also indicated that the bv truncation prevents the expression of functional Srrm4 protein in transfected HEK293 cells.

We also tested the functional status of Srrm4^bv^ *in vivo*, using zebrafish as an animal model. The endogenous Srrm4 mRNA of zebrafish (zSrrm4) was knocked down by injecting a previously described zSrrm4 morpholino (MO) [28] into fish eggs. Some of these eggs were also injected with either an mRNA encoding a MO-insensitive wild-type zSrrm4 (zSrrm4^wt^) or the zebrafish version of a MO-insensitive Srrm4^bv^ (zSrrm4^bv^). Three days later, the hair cells were visualized in the lateral line of zebrafish larvae using the fluorescent dye FM1–43 [43], [44]. We found that in the zSrrm4 MO-injected fish, the body axis was abnormally curved (Figure 5C versus Figure 5B, upper panel). This deformity in the body axis has previously been described for Srrm4 knock-down zebrafish, and has been attributed to neuronal defects [28]. In addition, we found that the number of hair cells was dramatically reduced in the zSrrm4 MO-injected fish (Figure 5C versus Figure 5B, lower panel, and quantitative analyses in Figure S10B and S10C). Co-injection of the zSrrm4^wt^ mRNA with the MO rescued both the body axis deformity and the hair-cell loss (Figure 5D), whereas co-injection of the zSrrm4^bv^ mRNA did not (Figure 5E, and statistical analyses in Figure S5B and S5C). These data suggest that Srrm4^bv^ is not functional, regardless of the expression system. Furthermore, our data show that although the loss of Srrm4 function has a broader phenotypic impact in zebrafish than in mice, Srrm4 is essential for hair-cell development in both species.

We hypothesized that a unique sequence motif may mark the Srrm4-regulated exons for splicing. Initially, we focused on exon sequences, testing a 9-nucleotide long exon whose splicing we had found to be Srrm4 regulated (*i.e. Dtna* exon 11). However, random mutation of 5 consecutive nucleotides in the 9-nucleotide exon did not affect its Srrm4-dependent splicing (). Next, we used the MEME software [45] to search for consensus motifs in both the Srrm4-regulated exons (n = 54) and the 50-nucleotide long portions of introns that are directly adjacent to these exons. MEME identified 3 motifs with *P*-values lower than 0.05, including a novel UGC motif and the known binding sequences of 2 splicing factors (*i.e.* U2af1 and the U1 small nuclear ribonucleoprotein). Alignment of the intron sequences upstream and downstream of the UGC motif showed that it is located near the 3′ end of the polypyrimidine tract (Figure 5F). To test whether UGC commonly occurs upstream of exons (i.e., regardless of Srrm4-dependent alternative splicing), we assessed all 50-bp regions that lie directly upstream of an exon in the Cacna1d and Ergic3 pre-mRNAs (n = 60). MEME did not detect UGC as a frequent motif in these sequences. Thus, UGC might be important for specifically Srrm4-dependent splicing.

Next, we used G-to-A point mutations to disrupt the selected UGC motifs in 6 minigenes that were randomly chosen from those that require Srrm4 for alternative splicing. In all cases, the mutations inhibited Srrm4-dependent exon inclusion (Figure 5G, Figure S11B–S11C). Mutagenesis experiments were also carried out to test the importance of the other two nucleotides in the UGC motif. We found that C-to-U substitutions (Figure 5G and Figure S11C), but not U-to-C/G/A mutations (data not shown) inhibited Srrm4-dependent exon inclusion. Thus, although a U nucleotide most often precedes the functionally relevant GC motif, only the GC nucleotides are required for Srrm4-dependent alternative splicing.

Blast searches revealed that the functionally relevant GC motifs are conserved among vertebrate species (Figure S11D). To test whether these motifs interact with Srrm4, we carried out streptavidin pull-down assays using two types of biotin-labeled RNA oligos. The ‘wild-type’ RNA oligo corresponded exactly to a 40-nucleotide long sequence around the splice acceptor site of the Srrm4-regulated exon in the Ergic3 pre-mRNA, whereas the ‘mutated’ RNA oligo contained a GC-to-AU substitution (Figure 5H). Western blot analysis of cell lysates prepared from flag-Srrm4-transfected HEK293 cells showed that only the ‘wild-type’ RNA efficiently pulled down flag-tagged Srrm4 (Figure 5H). These results suggest that conserved GC motifs upstream of the splice acceptor sites of Srrm4-regulated exons are necessary for the interaction between Srrm4 and the pre-mRNA.

## Discussion

In the present study, we show that the hearing and balance defects of *bv/bv* mice are caused by a mutation in the *Srrm4* gene. Using the bv mouse line and a genome-wide screening method to analyze the molecular function of Srrm4 *in vivo*, we identified Srrm4 as a key regulator of pre-mRNA splicing in the inner ear. Moreover, we found that Srrm4 was required for the alternative splicing of a specific set of exons that are marked by GC motifs near the 3′ ends of the polypyrimidine tracts. The *Srrm4* mutation in *bv/bv* mice also affected gene expression in the sensory patches of the inner ear, suggesting that Srrm4 controls a cascade of transcriptome-modifying events. Based on this analysis of the bv mouse line, we propose that Srrm4-regulated alternative splicing is critical for the differentiation of all sensory hair-cell types except the OHCs.

Although Srrm4 is expressed broadly in neural tissues, we did not detect splicing defects in the cerebellum and neocortex of P15 *bv/bv* mice. Moreover, if neurogenesis is impaired in *bv/bv* mouse embryos, the consequences of this defect are not readily detectable by Nissl staining at P15. Nevertheless, we cannot rule out the possibility that pre-mRNA splicing is affected at other time points or in other brain regions in these animals. Notably, a recent study showed that neurogenesis was impaired in E13/14 wild-type mice after neural progenitors in the ventricular zone were electroporated with vectors encoding an Srrm4-targeting shRNA [37]. Also, in examining the brains of bv mice, Matsuda and colleagues observed that immunofluorescence-based visualization of the parvalbumin-expressing GABAergic interneurons [46] detected abnormally few parvalbumin-expressing cells in the auditory cortex, somatosensory cortex, and anterior cingulate, whereas the visual cortex and the amygdala complex were unaffected. A possible interpretation of these data is that the Srrm4 defect in *bv/bv* mice directly affects the differentiation of interneurons in certain brain regions. Alternatively, some of the observed changes in the number of parvalbumin-expressing cells could be secondary to the hearing and balance defects in *bv/bv* mice. This possibility is consistent with the fact that congenital deafness has been shown to prevent the maturation of GABAergic transmission in the auditory cortex [47], [48], and sensory hearing loss has been associated with a decrease in the number of parvalbumin-positive cells in the superior olivary complex [49]. In addition, the lack of vestibular input has been reported to cause a reduction in the expression of various calcium-binding proteins, including parvalbumin, in the medial vestibular nucleus [50]. Thus, additional studies will be necessary to establish the etiology of the altered GABAergic interneuron density in certain brain regions of *bv/bv* mice.

In zebrafish, MO-mediated knock-down of Srrm4 has an obvious effect on both neural differentiation [28] and hair-cell development (Figure 5). Why does Srrm4 deficiency have a much greater impact on neural differentiation in zebrafish than in mice? One possible explanation is that splicing proteins other than Srrm4 have Srrm4-like functions in the mouse brain, but not in that of zebrafish. However, a more complex explanation is suggested by two findings. Firstly, whereas approximately 70% of the IHCs die between E18 and P5 in the bv mouse, this trend does not continue after P5 [27]. Secondly, the surviving IHCs are most likely functional because the *bv/bv* mice are not completely deaf. Together these data suggest that Srrm4 is not needed in the inner ear after a critical phase in development. This “critical phase” hypothesis is supported by the gene expression profile of the splicing suppressor polypyrimidine tract binding protein 1 (PTBP1), which has been shown to inhibit the constitutive inclusion of at least some Srrm4-regulated exons in Neuro2A cells [28]. PTBP1 is expressed in neural cells only during the early phases of differentiation [51]. Thus, Srrm4 may not be needed during the later phases of development when neural cells no longer contain PTBP1. We speculate that although Srrm4 deficiency could possibly lead to splicing defects in the neurons of both mice and zebrafish during early development, Srrm4-independent regulatory mechanisms are sufficient to support neuron differentiation until the end of the critical phase in mice but not in zebrafish.

Our finding that Srrm4-dependent exon inclusion requires the presence of a GC motif near the 3′ end of the polypyrimidine tract suggests that this motif serves as a *cis*-regulatory element for Srrm4-dependent splicing. *Cis*-regulatory elements are short sequence motifs that recruit RNA-binding proteins [52]; they can either enhance or suppress exon inclusion depending on which splicing factors are recruited and – in some cases – the position of the *cis*-regulatory element relative to the exon [53], [54]. Pre-mRNAs co-regulated by the same RNA-binding protein usually contain the same *cis*-regulatory element. Thus, the presence of the same motif next to almost every affected exon in the Srrm4 mutant mouse suggests that the inclusion of these exons into the mRNA is regulated by the same Srrm4-dependent mechanism.

Our RNA pull-down experiment suggested that the GC motif is necessary for the interaction between Srrm4 and the RNA. Whether this interaction is direct or mediated through other proteins remains to be determined. Notably, the GC motif is not the only sequence in the pre-mRNA that is important for the regulation of Srrm4-dependent splicing events. A previous study showed that pyrimidine-rich motifs are often present in introns that flank Srrm4-regulated exons [28], and that these pyrimidine-rich motifs are binding sites for PTBP1 [28]. Because the GC motifs are located near the 3′ end of the polypyrimidine sequences, it is tempting to speculate that the recruitment of either Srrm4 or Srrm4-binding proteins to the pre-mRNA may interfere with the binding of PTBP1.

**Figure 6:**
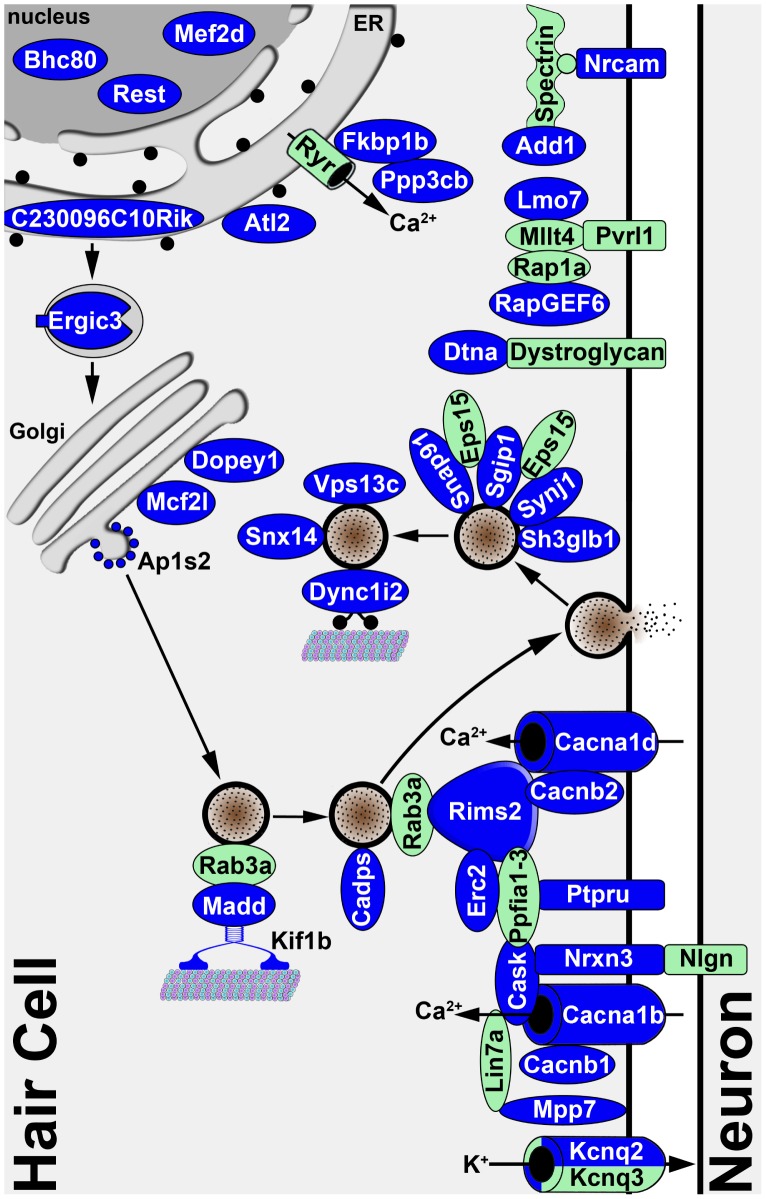
Diagram illustrating the known and predicted subcellular localizations of proteins encoded by validated Srrm4-regulated mRNAs. The proteins encoded by the validated mRNA targets of Srrm4-dependent splicing are shown in blue. The references for the protein localization and interaction data are listed in Table S3. For 8 of the proteins encoded by Srrm4-regulated mRNAs, the subcellular localization either falls outside of the depicted area or is unknown. Proteins that are not known to be regulated by Srrm4 but link Srrm4-regulated proteins to each other or a membrane are shown in green. Arrows indicate the direction of each transport event.

The fact that the Srrm4-regulated exons were found more frequently in the transcripts of proteins that are annotated with the GO terms ‘transmission of nerve impulse’ and ‘secretion by cell’ than in the transcripts of a random set of proteins suggests that the protein products of the Srrm4-regulated pre-mRNAs are functionally linked. We explored this possibility by collecting PubMed data on the subcellular localizations of, and interactions among, the affected proteins; given that we wanted to maximize the amount of information gathered, we did not restrict these PubMed searches to hair cell-related publications. Based on the information collected, we charted the likely subcellular localization of the affected proteins on a schematic model of the basolateral portion of a hair cell (Figure 6). This model illustrates that the majority of the proteins encoded by Srrm4-regulated transcripts may be associated with synaptic vesicles and the presynaptic plasma membrane. Notably, 42% of the proteins in this model that are encoded by Srrm4-regulated pre-mRNAs and have known protein-protein binding partners in the PubMed database interact with each other (see reference list in Table S3). Thus, both GO annotation analysis and the protein-protein interaction patterns suggest that the Srrm4-dependent modifications cluster predominantly in a single functional module of the proteome, and that this module is responsible for secretion and neurotransmission at the presynaptic side of the synapse. This analysis also suggests that the Srrm4-regulated proteins with uncharacterized molecular functions (*e.g.* Plekha6, 6330403A02Rik, and C230096C10Rik) are more likely involved in secretion or neurotransmission than in other biological processes.

Sustained high rates of neurotransmission from the IHCs and VHCs to their respective neural afferents require specialized presynaptic structures termed synaptic ribbons. Most OHCs contain synaptic ribbons only temporarily during differentiation; the only OHCs in which synaptic ribbons persist are those that are most apical [55]. Interestingly, in the *bv/bv* mice the OHCs are the only hair cells to remain intact, and many of the proteins with splicing defects are localized to synaptic ribbons (*i.e.* Cacna1d, Cask, Erc2, Rims2, Snap91, and Synj1 [56]). Thus, it seems plausible that the cell-type specificity of the synaptogenesis defect is due to an absence of protein isoforms that are specifically required for the formation of synaptic ribbons. Alternatively, it is possible that the mechanism that supports the inclusion of Srrm4-regulated exons in the cerebellum and neocortex of *bv/bv* mice also protects the OHCs from degeneration. These hypotheses could be tested by analyzing pre-mRNA splicing in the embryonic OHCs of *bv/bv* and control mice. However, RNA collection selectively from embryonic OHCs is technically challenging because of the physical proximity of OHCs and IHCs in the developing inner ear. Therefore, the analysis of pre-mRNA splicing in the OHCs of *bv/bv* mice is yet to be carried out.

Although we found that the majority of the splicing defects in the bv mouse line were associated with the secretory and synaptic apparatuses, the alternative splicing of at least two ciliary protein-encoding mRNAs (*i.e.* Bbs9 and Wdr35) were also altered. In addition, the *Srrm4* mutation led to reduced expression of the receptor-like inositol phosphatase Ptprq, which is required for the development of stereociliary bundles in the cochlea [57]. Srrm4-dependent splicing also affected at least 3 mRNAs that encode nuclear proteins (*i.e.* Rest, Bhc80, and Mef2d). Two of these (*i.e.* Rest and Bhc80) have been shown to have opposing effects on gene expression and have been reported to control vesicle processing and exocytosis through translational regulation [41], [58], [59]. We found that the genes regulated by Rest- and Bhc80 – but not those regulated by Mef2d – were overrepresented among the 44 whose expression was most reduced in the vestibular macula of *bv/bv* mice. Thus, the Srrm4-dependent splicing of the Rest and Bhc80 pre-mRNAs supports our hypothesis that Srrm4 plays a role in maturation of the regulated secretory apparatus in hair cells. Altered splicing of the Rest mRNA and reduced expression of the Rest target genes in the context of reduced Srrm4 function were described previously in Neuro2A cells subjected to RNA interference [37]. Thus, both *in vivo* and *in vitro* data suggest that the loss of Srrm4 function leads to a cascade of transcriptome alterations that affect both pre-mRNA splicing and gene expression. Further studies defining the importance of individual Srrm4-regulated exons in hair-cell development will enable us to elucidate the detailed pathogenesis of hair-cell degeneration in *bv/bv* mice.

In summary, in analyzing the bv mouse line we have identified Srrm4 as a regulator of alternative splicing that is required for the differentiation of hair cells in the hearing and balance organs. We propose that a Srrm4-regulated cascade of transcriptome modifying events adjusts the proteome of differentiating hair cells such that they take on neuron-like functions. Our study adds alternative splicing to the list of mechanisms that are critical for hair-cell differentiation. Given that some deafness-causing mutations are known to be localized to alternative exons (*i.e.* R643X in *PCDH15* [60] and R500X in *TRIC* [61]), understanding the regulation of alternative exon choice in the inner ear is expected to create therapeutic opportunities for the prevention of deafness.

## Methods

### Genetic analysis of the bv mouse line

The bv mouse strain was recovered from cryopreserved sperm samples (obtained from the European Mutant Mouse Archive), by intracytoplasmic sperm injection. All experiments and procedures were approved by the Animal Care and Use Committee of the University of Iowa. For mutation analysis, RNA was isolated from the inner ear of *bv/bv* and wild-type mice (E16.5), using the Trizol reagent. The coding regions of candidate mRNAs were amplified from the RNA samples using RT-PCR (see primers in Table S4), and the PCR products were sequenced. To amplify and sequence the genome adjacent to the deletion site in *bv/bv* mice, “genome walks” were carried out using the PCR-based Genome Walker Universal kit (Clontech Laboratories, Inc.) and a gene specific primer that anneals to the penultimate exon in *Srrm4* (5′-ACGGGACCTAAAGTATGGTGAGAAAG-3′). For genotyping, the presence or absence of the *bv* mutation was detected by PCR using tail DNA extracts, and 2 sets of primer pairs (wild-type allele: 5′-GGGAAGAGGTGGAGTATGTTG-3′ and 5′-CCTCGTGCTGGCATAGCTTTC-3′; bv allele: 5′-GAAAGAACCACAGCCCCGAGAA-3′ and 5′-CTGGGCAGGAGGGTACTTCTATAC-3).

### Generation and analysis of Srrm4 transgenic mice

The *Myo7a-Srrm4* transgene was constructed by subcloning the mouse Srrm4-encoding cDNA downstream of the mouse *Myo7a* promoter and upstream of the SV40 polyadenylation site in the pSTEC-1 vector, using standard PCR and subcloning methods (see PCR primers in Table S5). The Myo7a-Srrm4 expression cassette was isolated from pSTEC-1 by restriction digestion, and sent to Xenogen Corp. for the production of transgenic mice. The ABR thresholds of mice were measured at P21–28, using a previously described open-field system and broadband click stimuli [62]. The ability of mice to balance (P70–80) was evaluated by measuring the time each mouse could remain on a fixed horizontal rod (1.8 cm in diameter) following two training trials. Actin and Myo7a staining of whole-mount preparations of PFA-fixed cochlear and vestibular tissues was carried out as previously described [63], using the following reagent and antibodies: Alexa-488 labeled phalloidin (Invitrogen Corp.), rabbit anti-Myo7a antibody (Proteus Biosciences, Inc.), and Alexa-594 labeled anti-rabbit IgG (Invitrogen Corp.).

### *In situ* hybridization

Digoxigenin-labeled antisense exon 13 probe (coding nucleotides 1521–1827 in Srrm4), sense and antisense Srrm4 riboprobes (coding nucleotides 23–188) were generated using the DIG RNA Labeling Mix (Roche), and hybridized to inner ear samples of mice of various genotypes as described previously [17].

### Laser-capture microdissection and RNA extraction for microarray analysis

Inner ears of mouse embryos (E16.5) were embedded in Tissue-Tek O.C.T. Compound (Sakura Finetech, Inc.), frozen in liquid nitrogen, and cryosectioned. Sections were further processed for laser-capture microdissection using the Arcturus Histogene Frozen Section Staining kit (Applied Biosystems). The manufacturer's staining protocol was modified in that RNAse inhibitor (ProtectRNA, Sigma) was added to every solution in the kit that contains more than 5% water. The vestibular macula was captured from the inner ear sections using the Laser Capture Microdissection system (Pixcell II, Arcturus, Mountain View, CA). RNA was isolated from the captured tissue using the PicoPure RNA isolation kit. RNA was also extracted from the cerebellums of mice at P15, using the Trizol Reagent (Invitrogen). The cerebellar RNA was treated with DNase and further purified using the RNeasy mini kit (Qiagen).

### Microarray analysis

RNA samples for microarray analysis were processed using the NuGEN WT-Ovation Pico RNA Amplification System, NuGEN WT-Ovation Exon Module, and NuGEN FL-Ovation cDNA Biotin Module. Samples were hybridized to Mouse Exon Junction Microarrays (MJAY, Affymetrix Inc.). MJAY were scanned with an Affymetrix Model 7G upgraded scanner, and data were collected using GeneChip Operating Software. Raw microarray CEL files were imported into Partek Genomics Suite (Partek, Inc.). Signal intensities for the probe sets were quantile normalized and median polished using Robust Multichip Average background correction. The signal intensities of exon probe sets were used to calculate the overall expression level of each gene represented in MJAY. Normalized probe-set intensities (*I*_norm_) were calculated by dividing the background-corrected signal intensities of exon and exon-junction probe sets by the background-corrected gene-expression signal of the corresponding gene. The *I*_norm_ in the *bv/bv* and *bv/+* samples was analyzed by two-tailed Student *t*-test. Probe sets with significantly different *I*_norm_ (*P*<0.05) were queried against the Affymetrix annotation map file (which contains alternative/constitutive annotations for each measured splicing event) using simple Visual Basic for Application scripts, and probe sets that measure constitutive events were filtered out. The remaining probe sets were queried against the “SIB Alt-Splicing track” in the UCSC Genome Browser to identify and eliminate those that show either more than 50% identity with more than one gene or measure alternative promoter activity. The sequences of the remaining probe sets were queried against the mouse genome to identify those that measure the same splicing events. We required that probe sets targeting competing isoforms have opposite *I*_norm_ trends.

### RT–PCR and minigene-based validation of MJAY data

RT-PCR was carried out essentially as described previously [64]. We defined an alternative exon as ‘differently spliced’ in the *bv/bv* and *bv/+* samples if the RT-PCR data indicated that the inclusion rates for the exon were at least 1.5-fold different between the compared samples (Table S6 contains the inclusion rates calculated based on the RT-PCR data shown in Figure 3D and 3F, Figures S4, S7, and S8). Table S7 lists all primers that were used to generate the data shown in Figure 3D and 3F, Figures S4, S7, and S8.

For minigene-based validation of Srrm4-dependent splicing events, alternative exons and adjacent ∼300 bp intronic sequences were PCR amplified and subcloned into the exon trap pET-01 vector (Mobitec, see primers in Table S5). Mouse Srrm4^wt^ and Srrm4^bv^ were amplified by RT-PCR (see primers in Table S5) from inner ear RNA and subcloned into the pcDNA3.1 expression vector. The Srsf1 expression construct (Addgene plasmid 17990) has been described previously [65]. The minigines, Srrm4-encoding constructs, and the Srsf1-encoding plasmid were transfected into HEK293 cells using the Lipofectamine LTX and PLUS reagents (Invitrogen), and RNA was extracted from the cells 24 hours later using the RNeasy mini kit (Qiagen). RNA was reverse transcribed with Superscript III, and analyzed by RT-PCR using primers that annealed to the constitutive exons (primers: 5′-CACTTGGTGGAAGCTCTCTACC-3′ and 5′-CCACCTCCAGTGCCAAGGTC-3′). Site-directed mutagenesis of minigenes was carried out using overlap-extension PCR.

### Srrm4 knock-down and rescue experiments in zebrafish

The Srrm4 knock-down experiments were carried out in a transgenic zebrafish line developed by Haas and Gilmour [66]. In the neuromasts of these transgenic zebrafish, the claudin B promoter drives the expression of a membrane-tethered GFP (Tg[CldnB-mGFP]). zSrrm4 expression was knocked down by injecting the transgenic zebrafish (2-cell stage) with a previously described zSrrm4 MO [28] (5′-TTCTCCCAAAAGTACGCCAGCCATG-3′ from Gene Tools, Philomath, OR; 5 ng zSrrm4 morpholino/embryo). Since injection of 5 ng of the zSrrm4 MO led to non-specific toxicity, a p53-targeting MO (5′-GCGCCATTGCTTTGCAAGAATTG-3′ from Gene Tools; 5 ng/embryo) was co-injected. 3 days after injection, zebrafish larvae were incubated with 3 µM FM1–43 dye for 30 s to label the mechanosensing hair cells in the neuromasts. The FM1–43 staining led to a bright green signal that was much more intense than the GFP signal of the *CldnB-mGFP* transgene. After staining with FM1–43, the zebrafish were rinsed, anesthetized (0.02% 3-aminobenzoic acid ethyl ester), mounted in 3% methylcellulose, and photographed. The zSrrm4^wt^ and zSrrm4^bv^ mRNAs used for rescue experiments were generated using the mMessage mMachine kit (Ambion) and CS2+ plasmids that contained the zSrrm4^wt^ and zSrrm4^bv^ cDNAs (see cloning primers in Table S5). The zSrrm4^wt^ and zSrrm4^bv^ mRNAs were injected into zebrafish embryos (4-cell stage, 10 ng mRNA/embryo) that had previously been injected with MOs targeting zSrrm4 and p53. After the embryos had been maintained for 3 days, the mechanosensing hair cells were stained using the FM1–43 dye as described above.

### Western blotting

Nuclear fractions were isolated from the vestibular maculas of 44 wild-type and 44 *bv/bv* mice, on E16.5, using the Nuclear Complex Co-IP kit (Active Motif) according to the manufacturer's instructions. The obtained nuclear fractions were treated with Enzymatic Shearing Cocktail (Active Motif) and centrifuged at 16,000 g for 15 min at 4°C. The pellets were dissolved in SDS sample buffer, boiled for 3 min, resolved by SDS-PAGE, and electroblotted onto nitrocellulose membranes. Following a blocking incubation step, goat anti-Srrm4 antibody (sc-139291 from Santa Cruz Biotechnology Inc.) diluted 1∶200 or rabbit anti-Lamin B1 antibody (ab16048 from Abcam) diluted 1∶5,000 was added to the membranes for 14 hours. After multiple washing steps, membranes were incubated with HRP-conjugated secondary antibodies (anti-goat IgG and anti-rabbit IgG). Immunoblot signals were visualized using an Enhanced Chemiluminescence Detection System (Pierce Biotechnology).

### RNA pull-down assays

Flag-tagged Srrm4 was subcloned into the pcDNA3.1 expression vector and transfected into HEK293 cells. 24 hours after transfection, the cells were harvested and resuspended in buffer DG (containing 80 mM Potassium Glutamate, 0.1 mM EDTA, 10% glycerol, 0.01% NP40, 0.1 mM PMSF, 1 mM DTT, 16 µg/ml chymostatin, 10 µl/ml protease inhibitor cocktail [from Sigma], and 20 mM Hepes-KOH, pH 7.9). The cells were then sonicated, incubated on ice for 15 min, and centrifuged at 16,000 g for 15 min at 4°C. The supernatant was collected and diluted ∼5-fold in buffer DG supplemented with 2.2 mM MgCl_2_, 0.1 mg/ml tRNA (Invitrogen) and 1 U/ml RNase OUT. Mixtures of biotinylated RNA oligos (4 µg) and NutrAvidin agarose resin (∼45 µl from Pierce Biotechnology) were added to the cell lysates. Following 1.5-h incubation at 4°C, the resin was washed 6 times with buffer DG (supplemented with 2.2 mM MgCl_2_, 0.1 mg/ml tRNA, and 1 U/ml RNase OUT), resuspended in 45 µl of 2×SDS sample buffer, and boiled for 5 min. After a brief centrifugation, the supernatant fraction was resolved by SDS-PAGE, and protein was electroblotted onto nitrocellulose membranes. The membranes were blocked and incubated with 1∶1,000 dilution of a monoclonal anti-flag antibody (Sigma) for 14 hours. Following multiple washing steps, membranes were incubated with a HRP-conjugated secondary antibody (anti-mouse IgG). Signal was visualized with an Enhanced Chemiluminescence Detection System.

### Accession codes

Complete microarray datasets have been deposited at Gene Expression Omnibus under SuperSeries accession number GSE33591.

## Supporting Information

Figure S1 *In situ* hybridizations of sensory inner-ear regions of a wild-type mouse with a negative control probe. (A–C) Lack of signal in the cochlea (A), utricular macula (B), crista ampullaris (B), and saccular macula (C) of a wild-type mouse (P0), following *in situ* hybridization with a sense Srrm4 probe. OC: organ of Corti; U: utricle; AC: anterior crista; LC: lateral crista. Scale bars: 100 µm.
(PDF)

Figure S2 *In situ* hybridizations of sensory inner-ear regions of wild-type, *bv/bv*, and *Myo7a-Srrm4* transgenic *bv/bv* mice with an antisense probe corresponding to the coding region in *Srrm4* exon 13. (A–C) The wild-type mouse (P0) is positive for *Srrm4* exon 13 expression in the: cochlea (A), utricular macula (B), crista ampullaris (B), and saccular macula (C). (D–F) The *bv/bv* mouse (P0) is negative for *Srrm4* exon 13 expression in the cochlea (D), utricular macula (E), crista ampullaris (E), and saccular macula (F). (G–I) The *Myo7a-Srrm4* transgenic *bv/bv* mouse (P0) is positive for *Srrm4* exon 13 expression in the IHCs of the cochlea (G) and in the utricular macula (H), crista ampullaris (H), and saccular macula (I), but negative for expression in the OHCs (G); a lack of transgenic *Myo7a* promoter activity in OHCs has been observed in some *Myo7a-GFP* transgenic mouse lines [32]. OC: organ of Corti; U: utricle; AC: anterior crista; LC: lateral crista; IHC: inner hair cells. Scale bars: 100 µm.
(PDF)

Figure S3 Founder mouse-based breakdown of the ABR data and balance test results from Figure 3. (A) ABR thresholds of *bv/+*, *bv/bv*, and *Srrm4*-transgenic *bv/bv* mice on P21–28. (B) Time spent on a fixed horizontal rod before falling, by *bv/+*, *bv/bv*, and *Srrm4*-transgenic *bv/bv* mice on P70–80. Data obtained from progeny of a transgenic founder mouse are indicated with the same type of symbol. Each symbol represents the value for a single mouse. Mice used for the ABR and balance tests were derived from the breeding of pairs of *bv/+*:Tg and *bv/bv* mice.
(PDF)

Figure S4 Effect of the *Myo7a-Srrm4* transgene on the loss of sensory hair cells in *bv/bv* mice. (A–B) Counts of ciliated (A) utricular hair cells (HCs) and (B) cochlear IHCs in *bv/+*, *bv/bv*, and *Myo7a-Srrm4* transgenic *bv/bv* (*bv/bv*:Tg) mice (P5). Each symbol represents counts of utricular HCs or IHCs from a single mouse (one-way ANOVA, *P*<0.0001, post-hoc Tukey's test: *P<0.01, **P<0.001). (C) Organ of Corti preparations from *bv/+*, *bv/bv*, and *Srrm4*-transgenic *bv/bv* (*bv/bv:Tg*) mice (P28) were stained with phalloidin-Alexa Fluor 488 to visualize actin-rich structures including the stereocilia. Arrows indicate the row of IHCs. Scale bars: 20 µm.
(PDF)

Figure S5 Differences in the splicing of neuron-specific exons in the vestibular macula of *bv/bv* and *bv/+* mice. (A–B) RT-PCR analysis of alternative splicing in RNA samples extracted from laser-captured vestibular maculas of *bv/bv* and *bv/+* mice (E16.5). (A) Shown are amplified exons for which differences between the two genotypes resulted in *P*<0.05 for at least two MJAY probe sets per exon. The RT-PCR primers were designed to anneal to constitutive exons (white boxes) flanking the tested cassette exons (red boxes). In the case of non-cassette exons, arrows indicate the positions of primers that were used to test splicing. (B) RT-PCR amplified neuron-specific exons for which differences between the two genotypes resulted in *P*<0.05 for one MJAY probe set per exon. The numbers above the two mutually exclusive exons of the Cacna1b transcript indicate the length of the exons in bp. (C) RT-PCR testing of the tissue specificity of 13 randomly selected splicing events that were Srrm4-dependent in the vestibular macula. Cerebellum (ce) and spleen (sp) RNA samples were analyzed as examples of a neural and a non-neural RNA sample. (D–E) RT-PCR testing of the splicing of (D) nPTB exon 10 and (E) Rest exon 4 in the vestibular macula (vm), cerebellum (ce) and spleen (sp) of mice of the indicated genotypes. Rest exon 4, but not nPTB exon 10, is differently spliced in the vestibular maculas of *bv/bv* and *bv/+* mice (E16.5).
(PDF)

Figure S6 Fold differences in gene expression between the vestibular maculas of *bv/+* and *bv/bv* mice at E16.5. (A) Microarray data are shown for genes whose expression is increased or decreased at least 1.5-fold in the vestibular maculas of *bv/bv* mice *vs.* control (ctrl; *i.e. bv/+*) littermates. Cut-off for false discovery rate (FDR) was 0.15. Green shading indicates the genes known to be regulated by Rest. (B) Validation of gene expression differences between the *bv/bv* and control (ctrl; *i.e. bv/+*) vestibular maculas by real-time quantitative RT-PCR. The 6 tested genes were chosen from the list shown in panel A. The 18S rRNA was used for normalization purposes.
(PDF)

Figure S7 Evaluation of cerebellar histology and alternative splicing in *bv/bv* and *bv/+* mice. (A) Upper panel: RT-PCR results showing that the amount of the Srrm4 mRNA is higher in the cerebellum (ce) than that in the laser-captured vestibular macula (vm). Lower panel: RT-PCR results indicating that levels of the reference transcript (*i.e.* actin mRNA) are comparable in the two samples. (B) Nissl-stained parasagittal sections of the cerebellums of a *bv/+* (upper panels) and a *bv/bv* mouse (lower panels). Left panels: low-magnification images showing that the overall morphology of the cerebellar lobes is normal in the *bv/bv* mouse. Right panels: higher magnification images showing that the overall organization of the molecular, ganglionic, and granular cell layers is intact in the *bv/bv* mouse. Scale bars: 100 µm. (C) Microarray heat map of normalized probe-set signals calculated based on the results of a comparative MJAY analysis of cerebellar RNA samples from *bv/bv* and *bv/+* mice. The criterion for inclusion was that at least 2 probe sets per exon (connected by brackets) indicated that differences in the expression of alternative exons between the cerebellums of 4 *bv/+* and 4 *bv/bv* mice were significant. Dots at the left margin represent the data generated by exon-skipping probe sets. (D) RT-PCR experiments testing the validity of 15 of the 18 MJAY “hits” shown in panel C; this analysis reveals that those hits were false positives. RT-PCR results for the remaining 3 MJAY hits are not shown because these reactions did not generate PCR products. The RT-PCR primers were designed to anneal to constitutive exons (white boxes) flanking the tested cassette exons (red boxes). In the case of non-cassette exons, arrows indicate the positions of primers that were used to test splicing. The numbers above the two alternative exons of the Stx transcript indicate the length of the exons in bp. (E) RT-PCR analysis of the splicing of 20 exons in the cerebellums of *bv/bv* and *bv/+* mice. The tested exons were selected randomly from among those that are Srrm4-regulated in the vestibular macula.
(PDF)

Figure S8 RT-PCR evaluation of the alternative splicing of 10 selected exons in the neocortex of *bv/bv* and *bv/+* mice. (A) RT-PCR analysis of exon inclusion rates in the neocortex of *bv/bv* and *bv/+* mice (P15), for 8 exons selected based on a reduction in the inclusion rate for the vestibular macula of *bv/bv* mice shown in Figure 4D. The RT-PCR primers were designed to anneal to constitutive exons (white boxes) flanking the tested alternative exons (red boxes). (B) RT-PCR analysis of exon inclusion rates in the neocortex of *bv/bv* and *bv/+* mice (P15) for 2 exons that were randomly selected from among the previously identified Srrm4-regulated exons [28].
(PDF)

Figure S9 Evaluation of the brain histology of *bv/bv* and wild-type mice on P15. (A) Mouse brain preparation illustrating the coronal section planes used to analyze the central nervous system of +/+ and *bv/bv* mice. Capital letters next to the horizontal lines identify the regions from which coronal sections were prepared. (B–Q) Left panels: low-magnification images of Nissl-stained coronal sections from *+/+* (B–I) and *bv/bv* mice (J–Q). Rectangles labeled ‘m’ and ‘r’ indicate the regions that are shown at higher magnification (*i.e.* 10× objective) in the middle and right-hand panels. Middle panels: cortical regions of +/+ and *bv/bv* mice representing the ‘m’ areas indicated in the left-hand panels. Right-hand panels: cortical regions of +/+ and *bv/bv* mice representing the ‘r’ areas indicated in the left-hand panels.
(PDF)

Figure S10 Functional analysis of Srrm4^wt^ and Srrm4^bv^ in HEK293 cells and zebrafish. (A) RT-PCR testing of alternative splicing in HEK293 cells transfected with both a minigene and a protein-encoding expression vector. The expression vectors encoded Srrm4^wt^, Srrm4^bv^, Srsf1, or no protein (vector control). Each minigene contained an alternative exon (red box), adjacent intronic sequences (∼300 bp each), and a constitutive exon at each end (white boxes). The promoter and polyadenylation site (pA) of the minigene cassette are indicated. The RT-PCR primers (arrows) were designed to anneal to the constitutive exons. Results obtained with 12 minigenes are shown. (B–C) Statistical analysis of the number of (B) mGFP-positive neuromasts and (C) FM1–43-stained hair cells (HCs) in the following groups of *claudin B-mGFP* transgenic zebrafish (ZF; 72 hpf): non-injected (control, n = 20), zSrrm4 MO-injected (n = 25), zSrrm4 MO- and zSrrm4^bv^-injected (n = 9), and Srrm4 MO- and zSrrm4^wt^-injected (n = 16) (one-way ANOVA, *P*<0.0001, post-hoc Bonferroni's test: *P<0.01, **P<0.001; NS: non-significant).
(PDF)

Figure S11 The effects of exon and intron mutations on the Srrm4-dependent inclusion of alternative exons into the mature mRNA. (A) RT-PCR testing of alternative splicing of control (WT) and mutated (M) Dtna exon 11. For the RT-PCR tests, RNA was extracted from HEK293 cells that were transfected with both a Srrm4 construct (Srrm4^wt^, +; or empty pcDNA3.1 vector, −) and a Dtna exon 11-containing minigene (control, WT; or mutant, M). The sequence of the alternative exon is highlighted by blue characters; the mutated nucleotides are indicated by bolding. The minigenes also contained the exon-flanking intron sequences (300–300 bp) from *Dtna*. (B–C) Effects of mutations in selected UGC sequences on Srrm4-dependent splicing. RT-PCR testing of alternative splicing in HEK293 cells transfected with both an Srrm4 construct (Srrm4^wt^, +; or empty pcDNA3.1 vector, −) and a minigene consisting of exons and introns. The control (WT) minigenes did not contain mutations, whereas the mutant minigenes (M, or M1–3) contained base substitutions. The relevant sequence fragments for each encoded pre-mRNA are shown. Bolding indicates the mutated bases; hyphens indicate the intron-exon borders; levels of exon inclusion are shown in percentages. G-to-A (B,C) and C-to-U (C) substitutions in the encoded pre-mRNAs were designed to alter selected GC motifs. (D) Nucleotide conservation in the intronic regions located immediately upstream of Srrm4-regulated alternative exons. The heights of red columns indicate the vertebrate base-wise conservation scores calculated by PhyloP [67]. Positive numbers indicate conservation, negative values indicate accelerated evolution. The underlined GC motifs contain the G nucleotides that were found to be important for Srrm4-dependent splicing.
(PDF)

Table S1 Genomic sequences near the end of the *Srrm4* gene in wild-type and *bv/bv* mice.
(XLS)

Table S2 Exons showing abnormally low inclusion rates in the vestibular macula of *bv/bv* mice.
(XLS)

Table S3 Publications describing the subcellular localization and interactions of proteins encoded by Srrm4-regulated mRNAs.
(XLS)

Table S4 Primers used for the amplification of transcripts encoded by candidate genes at the *bv* locus.
(XLS)

Table S5 Primers used for the production of expression constructs.
(XLS)

Table S6 Inclusion rates of alternative exons in the vestibular macula (vm), cerebellum, and neocortex of *bv/+* and *bv/bv* mice in percentage.
(XLS)

Table S7 Primers used for the RT-PCR validation of microarray data.
(XLS)
