# Supplementary figures and images for: A Mutation in the Srrm4 Gene Causes Alternative Splicing Defects and Deafness in the Bronx Waltzer Mouse
Source: PLoS Genet. 2012 Oct 4;8(10):e1002966. doi: 10.1371/journal.pgen.1002966 (PMC3464207; doi:10.1371/journal.pgen.1002966)

Figure S1

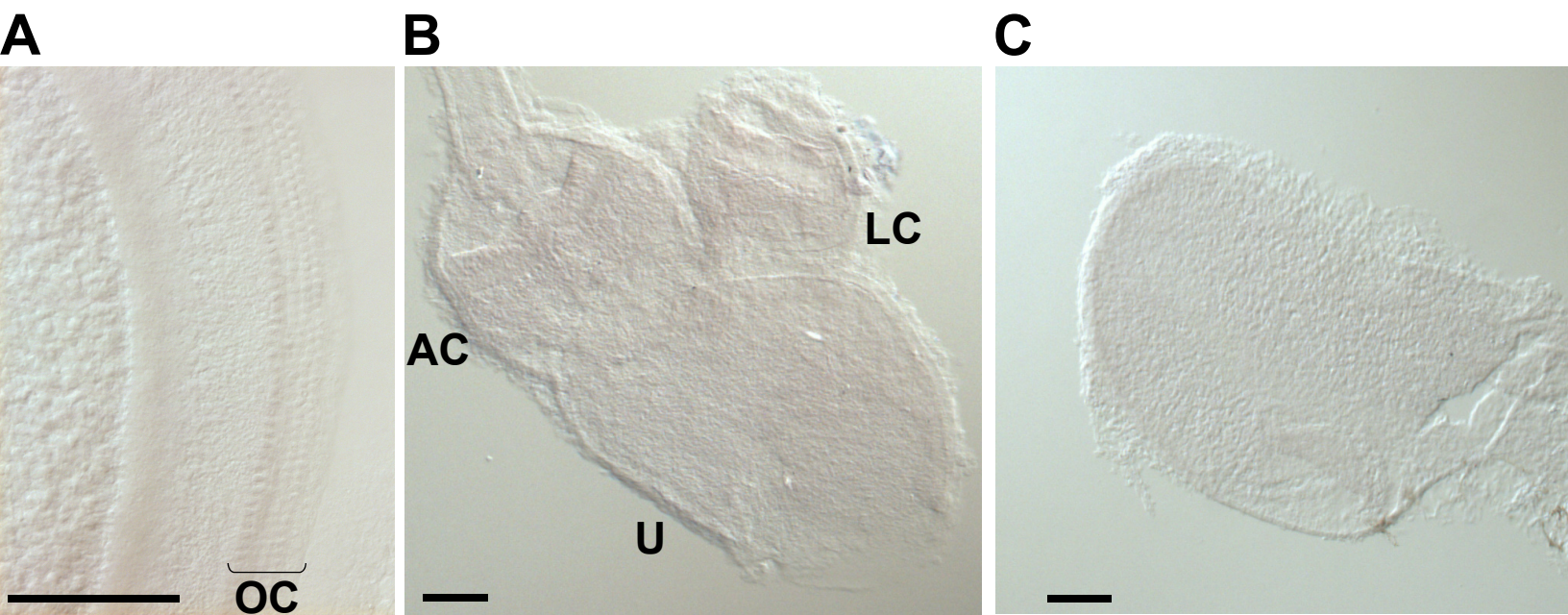

Supplement: Figure S1 — In situ hybridizations of sensory inner-ear regions of a wild-type mouse with a negative control probe. (A–C) Lack of signal in the cochlea (A), utricular macula (B), crista ampullaris (B), and saccular macula (C) of a wild-type mouse (P0), following in situ hybridization with a sense Srrm4 probe. OC: organ of Corti; U: utricle; AC: anterior crista; LC: lateral crista. Scale bars: 100 µm. (PDF) [file pgen.1002966.s001.pdf]

Figure S2

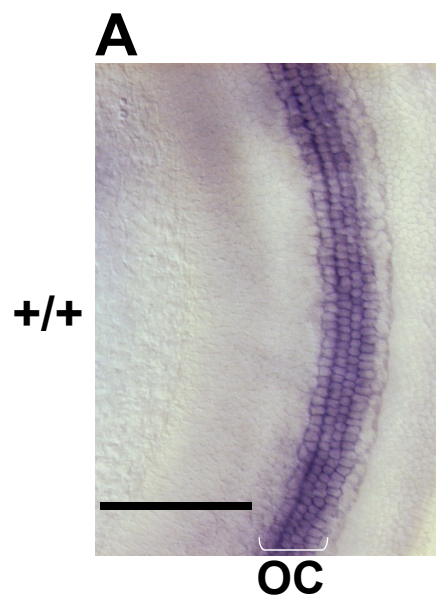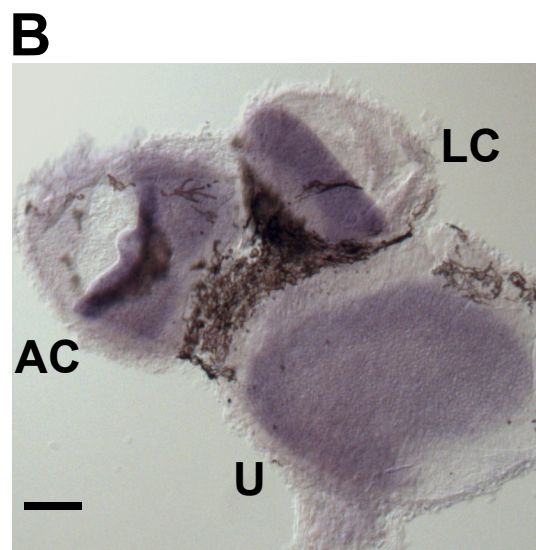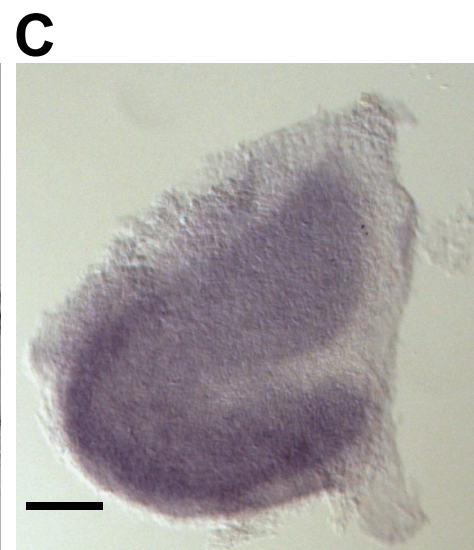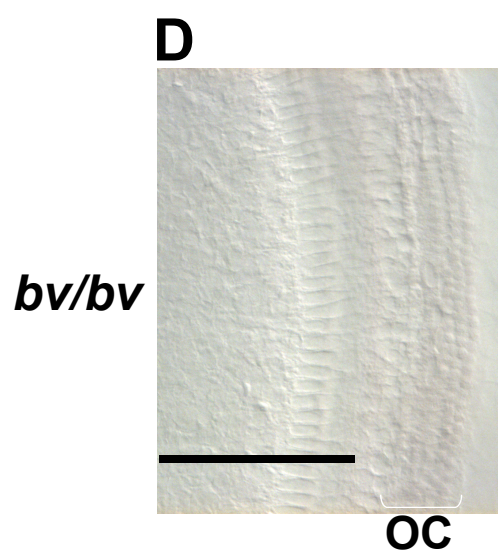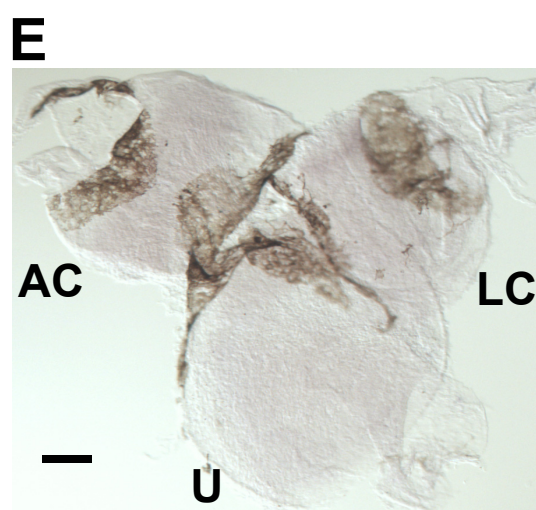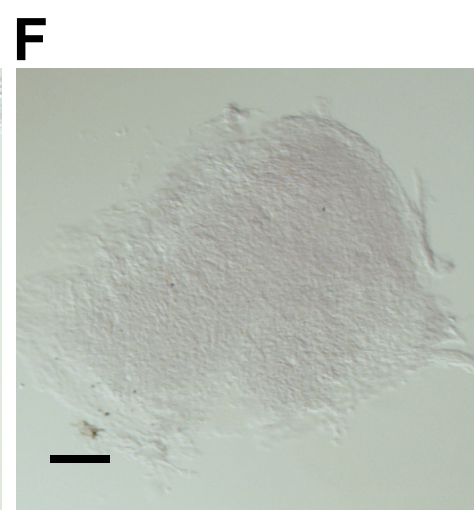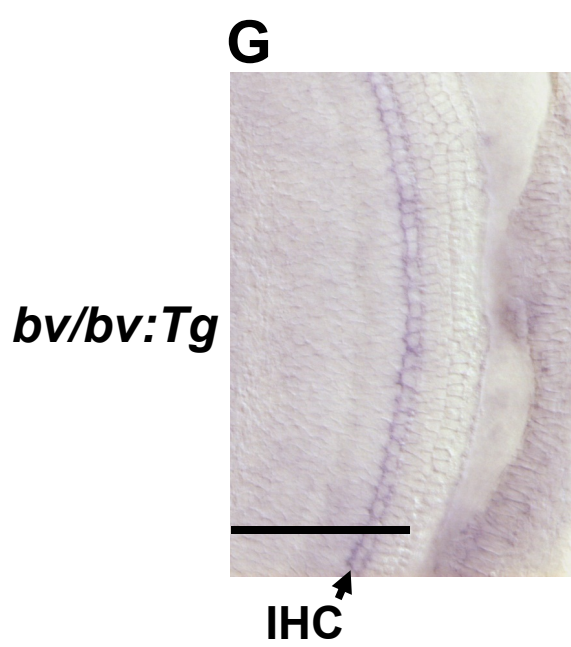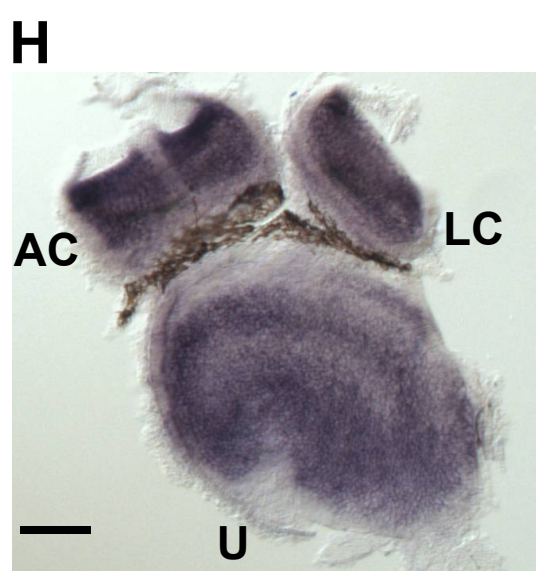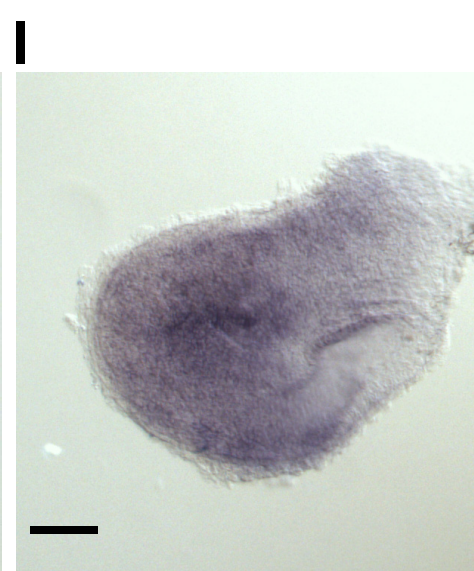

Supplement: Figure S2 — In situ hybridizations of sensory inner-ear regions of wild-type, bv/bv, and Myo7a-Srrm4 transgenic bv/bv mice with an antisense probe corresponding to the coding region in Srrm4 exon 13. (A–C) The wild-type mouse (P0) is positive for Srrm4 exon 13 expression in the: cochlea (A), utricular macula (B), crista ampullaris (B), and saccular macula (C). (D–F) The bv/bv mouse (P0) is negative for Srrm4 exon 13 expression in the cochlea (D), utricular macula (E), crista ampullaris (E), and saccular macula (F). (G–I) The Myo7a-Srrm4 transgenic bv/bv mouse (P0) is positive for Srrm4 exon 13 expression in the IHCs of the cochlea (G) and in the utricular macula (H), crista ampullaris (H), and saccular macula (I), but negative for expression in the OHCs (G); a lack of transgenic Myo7a promoter activity in OHCs has been observed in some Myo7a-GFP transgenic mouse lines [32]. OC: organ of Corti; U: utricle; AC: anterior crista; LC: lateral crista; IHC: inner hair cells. Scale bars: 100 µm. (PDF) [file pgen.1002966.s002.pdf]

**A**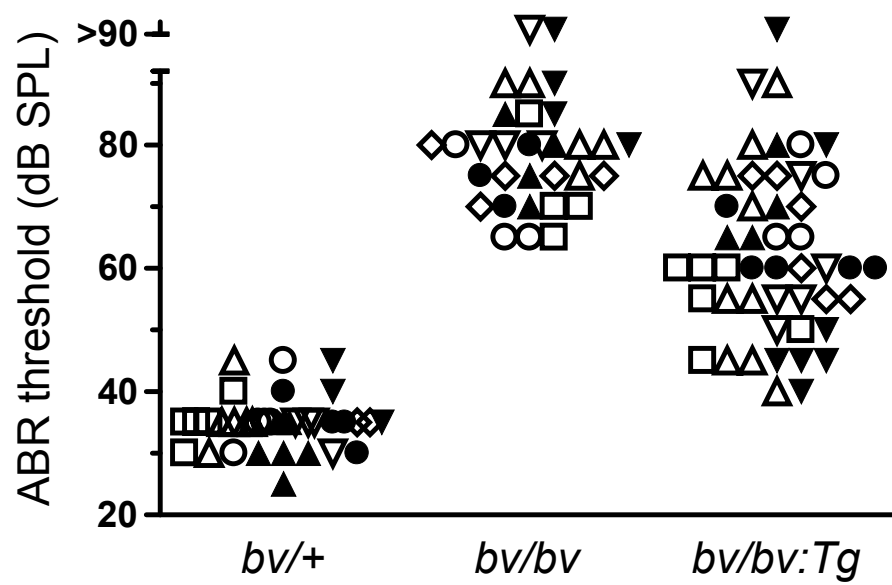**B**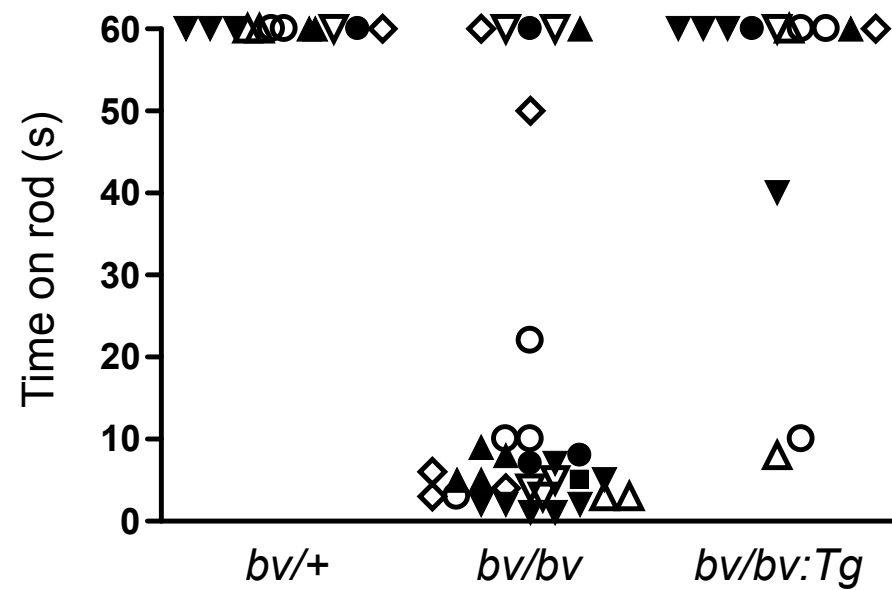

Supplement: Figure S3 — Founder mouse-based breakdown of the ABR data and balance test results from Figure 3. (A) ABR thresholds of bv/+, bv/bv, and Srrm4-transgenic bv/bv mice on P21–28. (B) Time spent on a fixed horizontal rod before falling, by bv/+, bv/bv, and Srrm4-transgenic bv/bv mice on P70–80. Data obtained from progeny of a transgenic founder mouse are indicated with the same type of symbol. Each symbol represents the value for a single mouse. Mice used for the ABR and balance tests were derived from the breeding of pairs of bv/+:Tg and bv/bv mice. (PDF) [file pgen.1002966.s003.pdf]

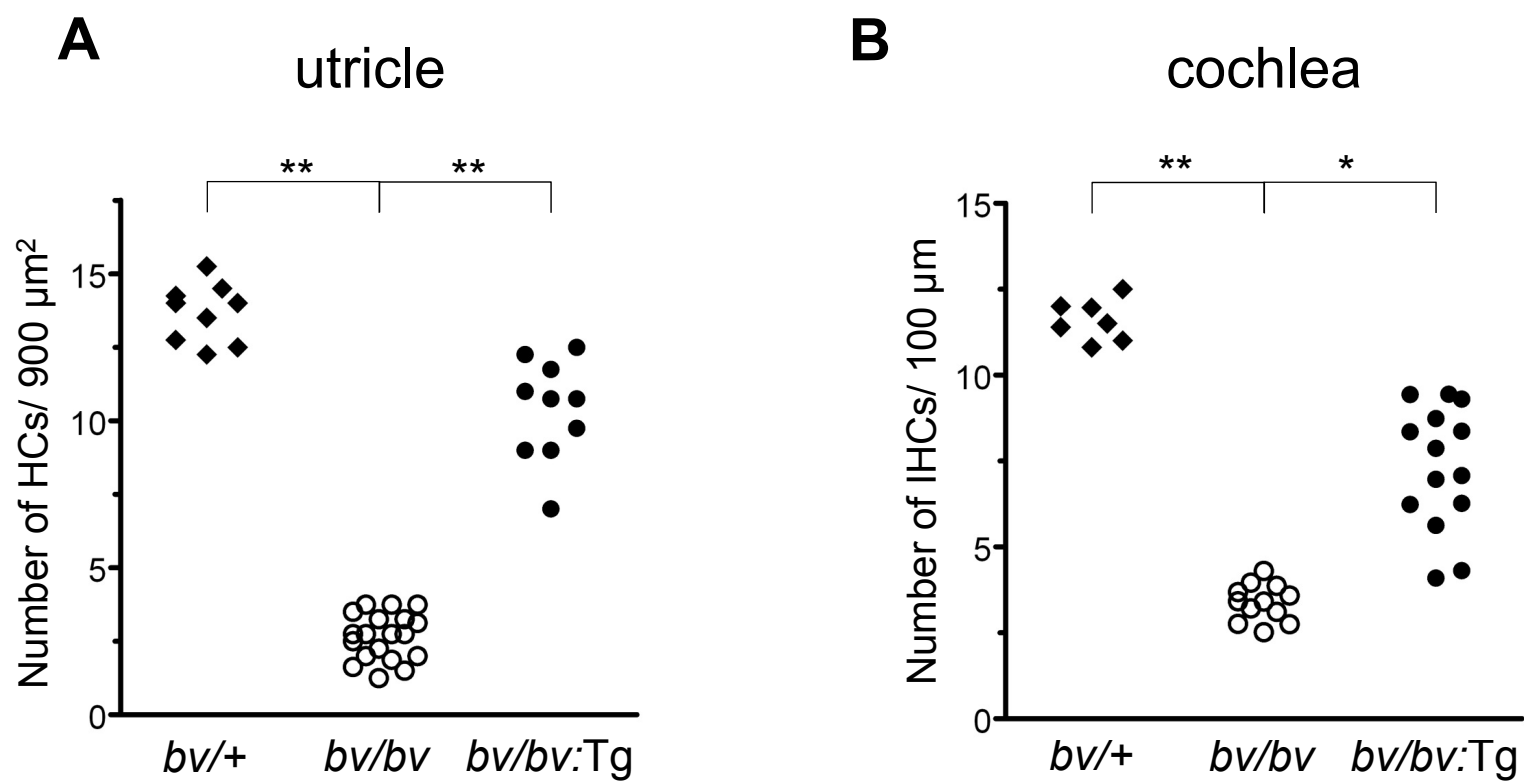**C**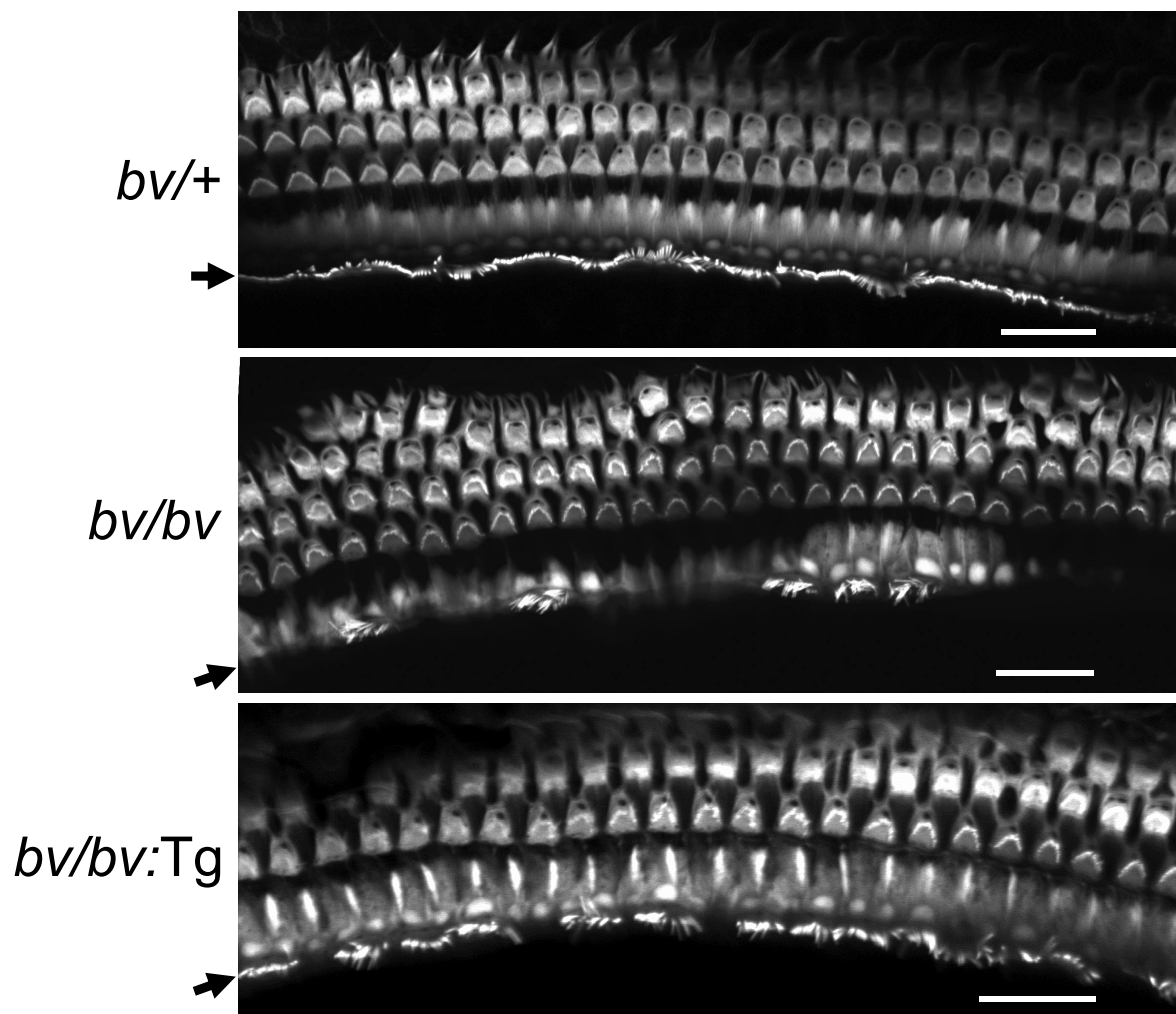

Supplement: Figure S4 — Effect of the Myo7a-Srrm4 transgene on the loss of sensory hair cells in bv/bv mice. (A–B) Counts of ciliated (A) utricular hair cells (HCs) and (B) cochlear IHCs in bv/+, bv/bv, and Myo7a-Srrm4 transgenic bv/bv (bv/bv:Tg) mice (P5). Each symbol represents counts of utricular HCs or IHCs from a single mouse (one-way ANOVA, P<0.0001, post-hoc Tukey's test: *P<0.01, **P<0.001). (C) Organ of Corti preparations from bv/+, bv/bv, and Srrm4-transgenic bv/bv (bv/bv:Tg) mice (P28) were stained with phalloidin-Alexa Fluor 488 to visualize actin-rich structures including the stereocilia. Arrows indicate the row of IHCs. Scale bars: 20 µm. (PDF) [file pgen.1002966.s004.pdf]

**A**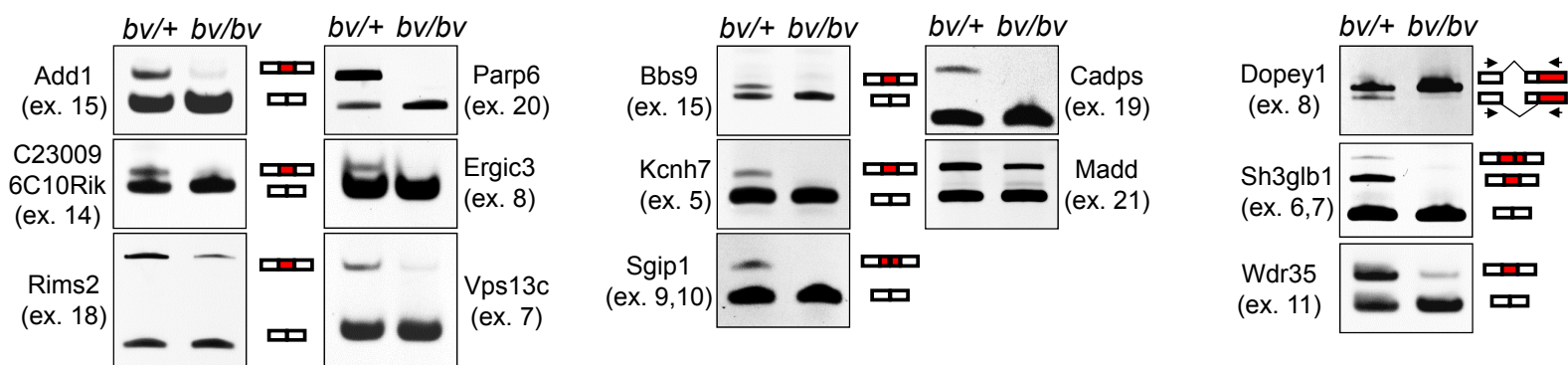**B**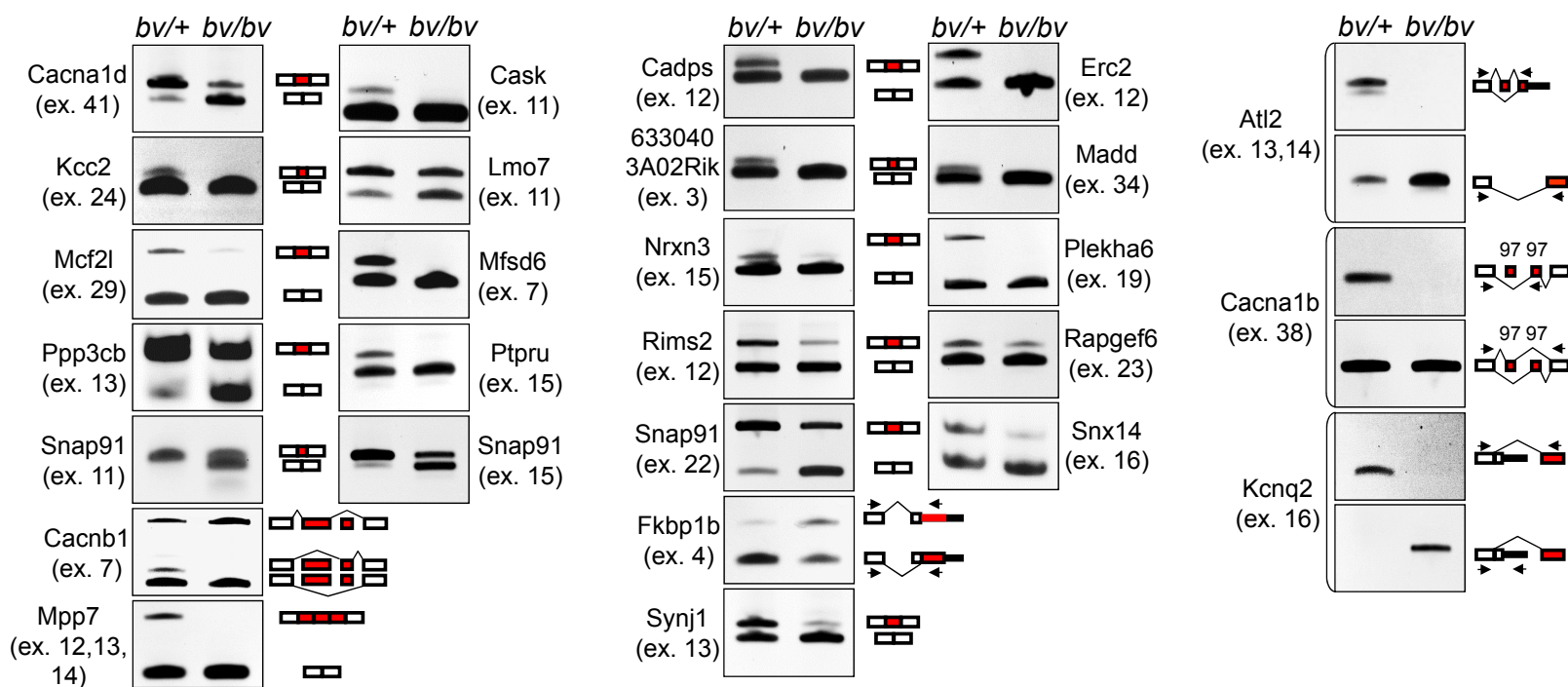**C**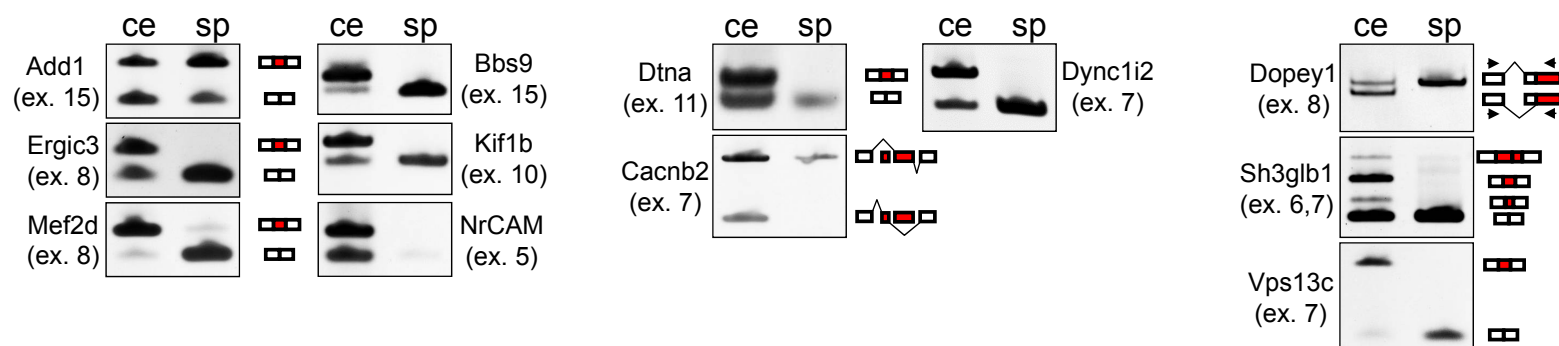**D**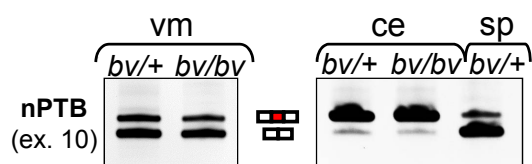**E**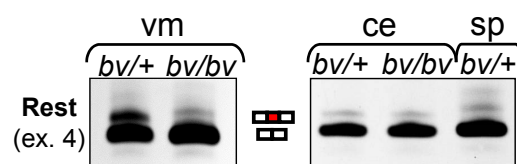

Supplement: Figure S5 — Differences in the splicing of neuron-specific exons in the vestibular macula of bv/bv and bv/+ mice. (A–B) RT-PCR analysis of alternative splicing in RNA samples extracted from laser-captured vestibular maculas of bv/bv and bv/+ mice (E16.5). (A) Shown are amplified exons for which differences between the two genotypes resulted in P<0.05 for at least two MJAY probe sets per exon. The RT-PCR primers were designed to anneal to constitutive exons (white boxes) flanking the tested cassette exons (red boxes). In the case of non-cassette exons, arrows indicate the positions of primers that were used to test splicing. (B) RT-PCR amplified neuron-specific exons for which differences between the two genotypes resulted in P<0.05 for one MJAY probe set per exon. The numbers above the two mutually exclusive exons of the Cacna1b transcript indicate the length of the exons in bp. (C) RT-PCR testing of the tissue specificity of 13 randomly selected splicing events that were Srrm4-dependent in the vestibular macula. Cerebellum (ce) and spleen (sp) RNA samples were analyzed as examples of a neural and a non-neural RNA sample. (D–E) RT-PCR testing of the splicing of (D) nPTB exon 10 and (E) Rest exon 4 in the vestibular macula (vm), cerebellum (ce) and spleen (sp) of mice of the indicated genotypes. Rest exon 4, but not nPTB exon 10, is differently spliced in the vestibular maculas of bv/bv and bv/+ mice (E16.5). (PDF) [file pgen.1002966.s005.pdf]

A

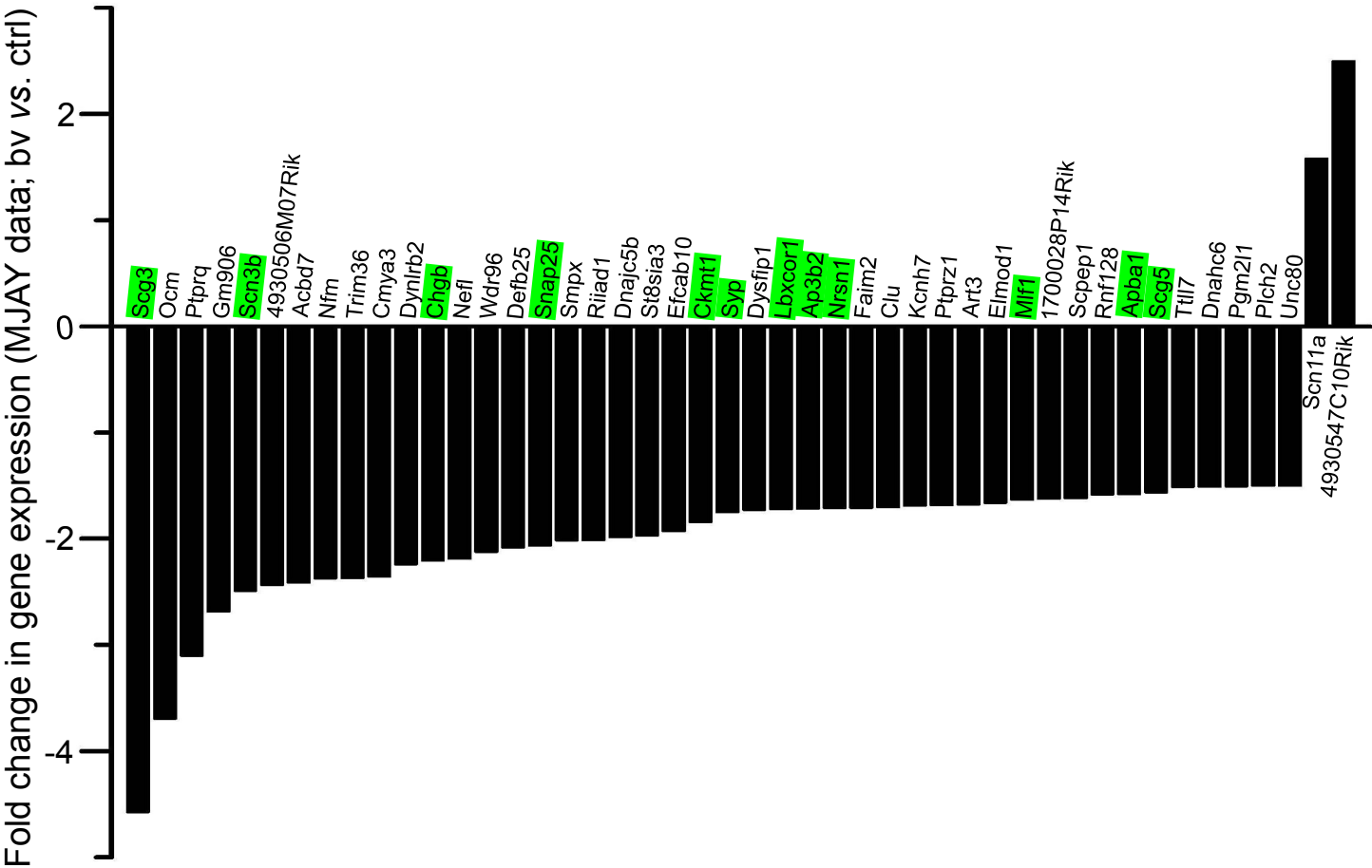

B

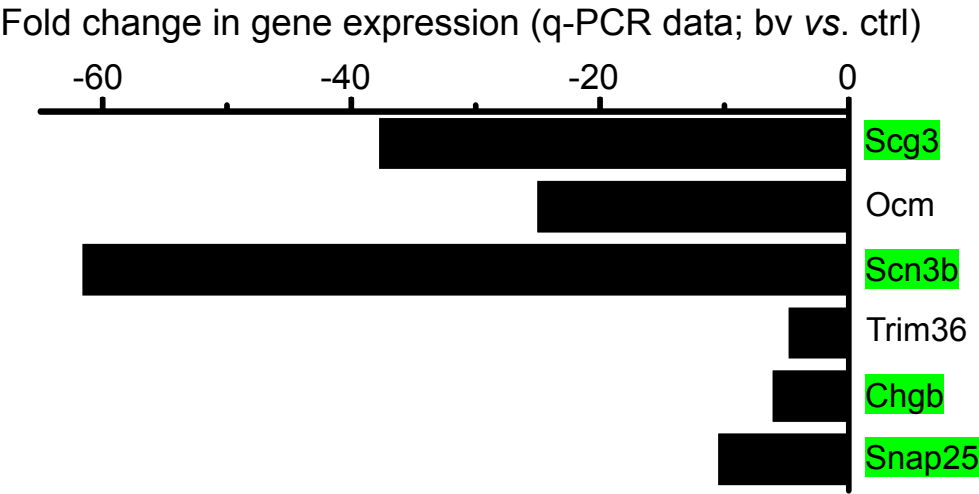

Supplement: Figure S6 — Fold differences in gene expression between the vestibular maculas of bv/+ and bv/bv mice at E16.5. (A) Microarray data are shown for genes whose expression is increased or decreased at least 1.5-fold in the vestibular maculas of bv/bv mice vs. control (ctrl; i.e. bv/+) littermates. Cut-off for false discovery rate (FDR) was 0.15. Green shading indicates the genes known to be regulated by Rest. (B) Validation of gene expression differences between the bv/bv and control (ctrl; i.e. bv/+) vestibular maculas by real-time quantitative RT-PCR. The 6 tested genes were chosen from the list shown in panel A. The 18S rRNA was used for normalization purposes. (PDF) [file pgen.1002966.s006.pdf]

**A**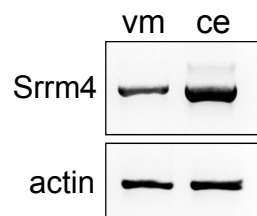**B**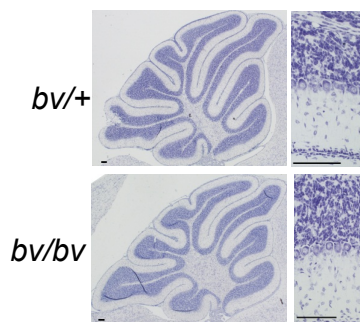**C**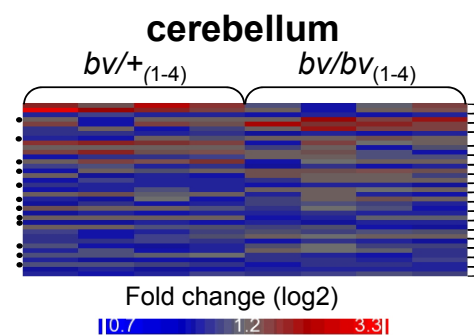**D**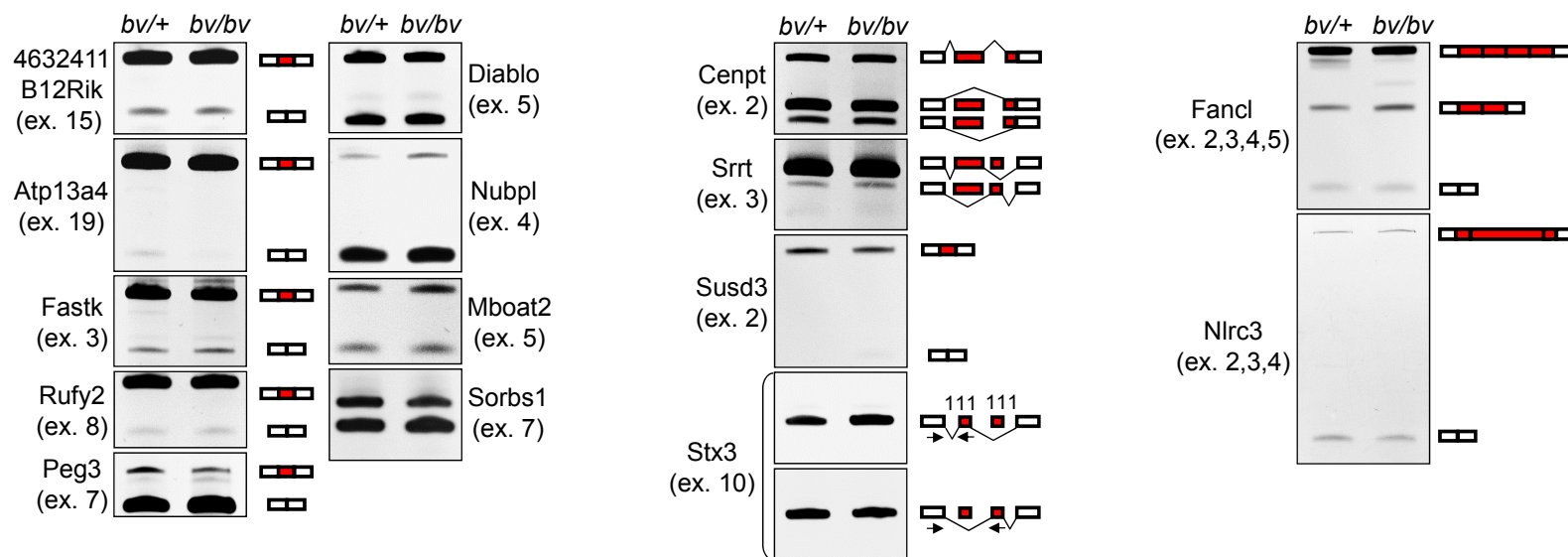**E**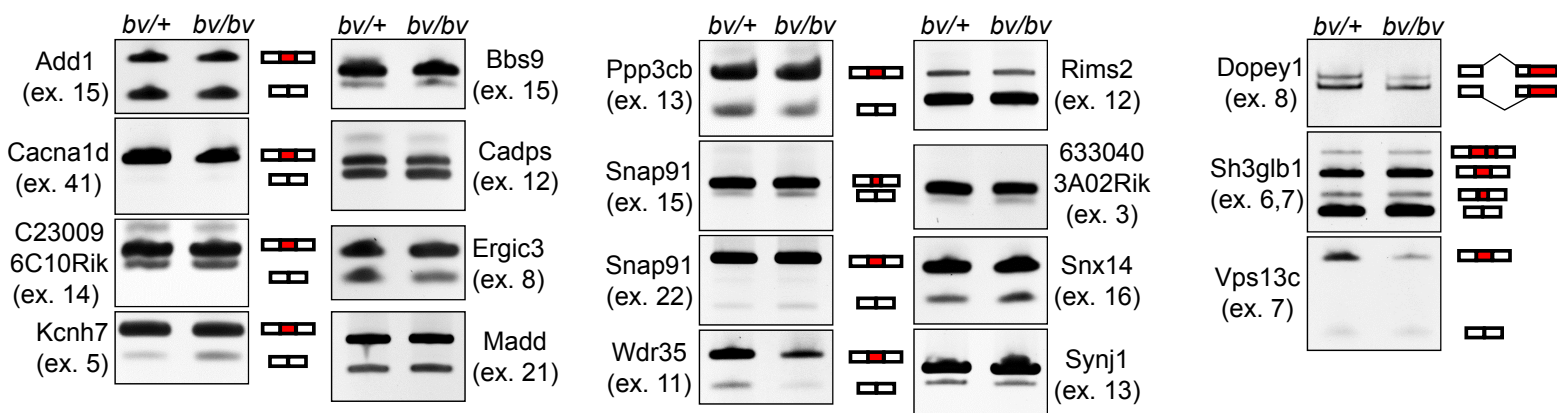

Supplement: Figure S7 — Evaluation of cerebellar histology and alternative splicing in bv/bv and bv/+ mice. (A) Upper panel: RT-PCR results showing that the amount of the Srrm4 mRNA is higher in the cerebellum (ce) than that in the laser-captured vestibular macula (vm). Lower panel: RT-PCR results indicating that levels of the reference transcript (i.e. actin mRNA) are comparable in the two samples. (B) Nissl-stained parasagittal sections of the cerebellums of a bv/+ (upper panels) and a bv/bv mouse (lower panels). Left panels: low-magnification images showing that the overall morphology of the cerebellar lobes is normal in the bv/bv mouse. Right panels: higher magnification images showing that the overall organization of the molecular, ganglionic, and granular cell layers is intact in the bv/bv mouse. Scale bars: 100 µm. (C) Microarray heat map of normalized probe-set signals calculated based on the results of a comparative MJAY analysis of cerebellar RNA samples from bv/bv and bv/+ mice. The criterion for inclusion was that at least 2 probe sets per exon (connected by brackets) indicated that differences in the expression of alternative exons between the cerebellums of 4 bv/+ and 4 bv/bv mice were significant. Dots at the left margin represent the data generated by exon-skipping probe sets. (D) RT-PCR experiments testing the validity of 15 of the 18 MJAY “hits” shown in panel C; this analysis reveals that those hits were false positives. RT-PCR results for the remaining 3 MJAY hits are not shown because these reactions did not generate PCR products. The RT-PCR primers were designed to anneal to constitutive exons (white boxes) flanking the tested cassette exons (red boxes). In the case of non-cassette exons, arrows indicate the positions of primers that were used to test splicing. The numbers above the two alternative exons of the Stx transcript indicate the length of the exons in bp. (E) RT-PCR analysis of the splicing of 20 exons in the cerebellums of bv/bv and bv/+ mice. The tested ex [file pgen.1002966.s007.pdf]

**A**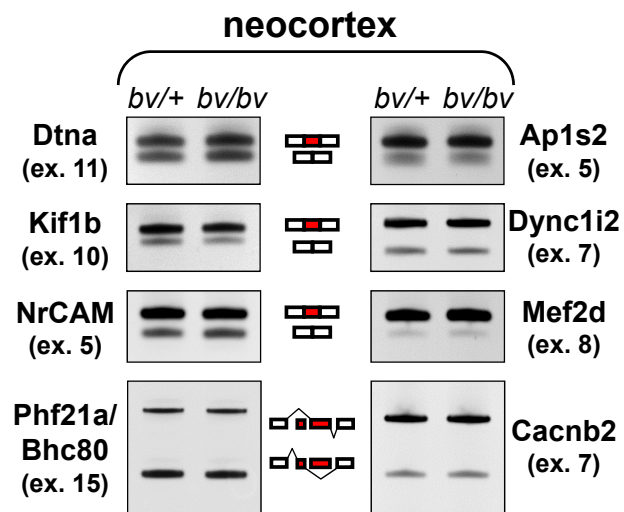**B**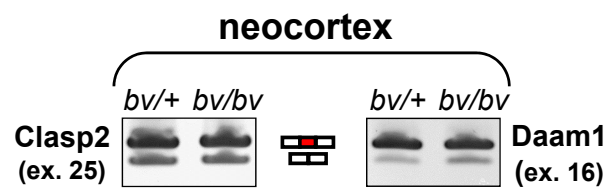

Supplement: Figure S8 — RT-PCR evaluation of the alternative splicing of 10 selected exons in the neocortex of bv/bv and bv/+ mice. (A) RT-PCR analysis of exon inclusion rates in the neocortex of bv/bv and bv/+ mice (P15), for 8 exons selected based on a reduction in the inclusion rate for the vestibular macula of bv/bv mice shown in Figure 4D. The RT-PCR primers were designed to anneal to constitutive exons (white boxes) flanking the tested alternative exons (red boxes). (B) RT-PCR analysis of exon inclusion rates in the neocortex of bv/bv and bv/+ mice (P15) for 2 exons that were randomly selected from among the previously identified Srrm4-regulated exons [28]. (PDF) [file pgen.1002966.s008.pdf]

Figure S9

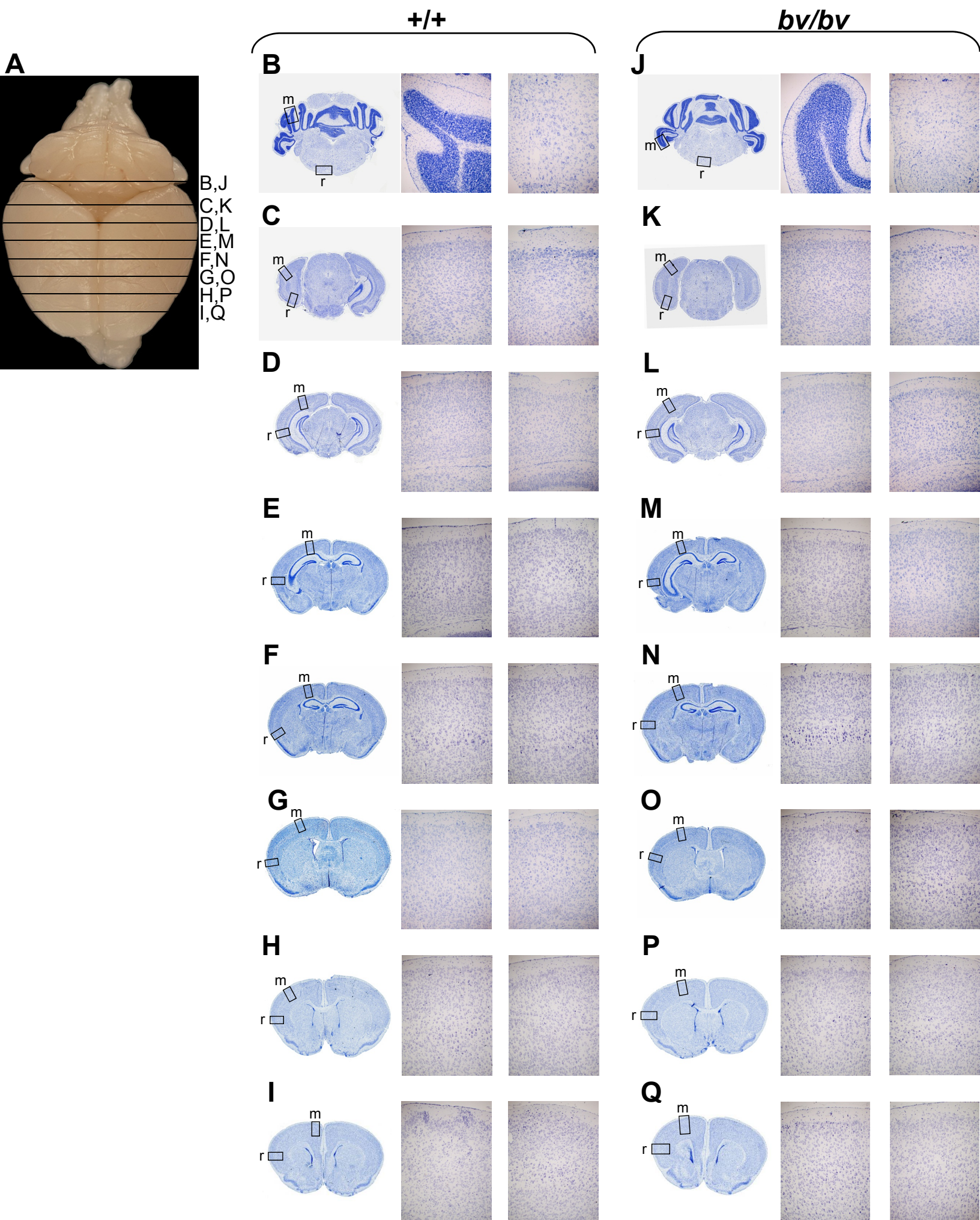

Supplement: Figure S9 — Evaluation of the brain histology of bv/bv and wild-type mice on P15. (A) Mouse brain preparation illustrating the coronal section planes used to analyze the central nervous system of +/+ and bv/bv mice. Capital letters next to the horizontal lines identify the regions from which coronal sections were prepared. (B–Q) Left panels: low-magnification images of Nissl-stained coronal sections from +/+ (B–I) and bv/bv mice (J–Q). Rectangles labeled ‘m’ and ‘r’ indicate the regions that are shown at higher magnification (i.e. 10× objective) in the middle and right-hand panels. Middle panels: cortical regions of +/+ and bv/bv mice representing the ‘m’ areas indicated in the left-hand panels. Right-hand panels: cortical regions of +/+ and bv/bv mice representing the ‘r’ areas indicated in the left-hand panels. (PDF) [file pgen.1002966.s009.pdf]

**A**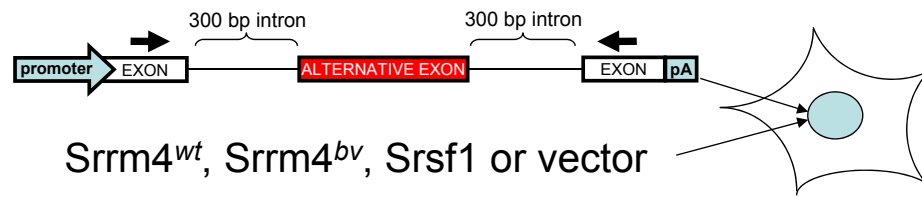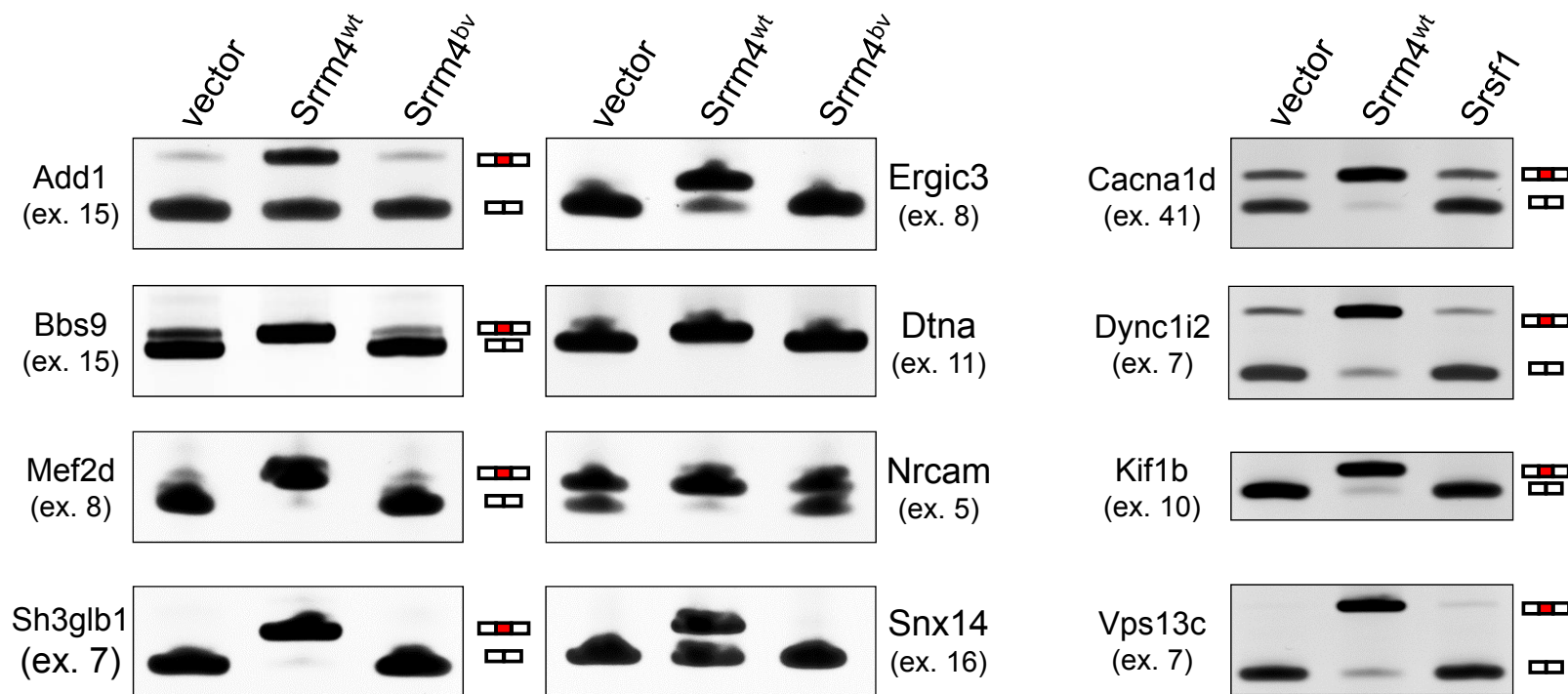**B**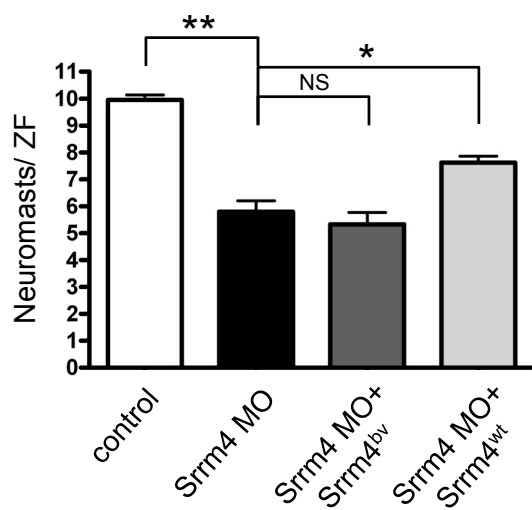**C**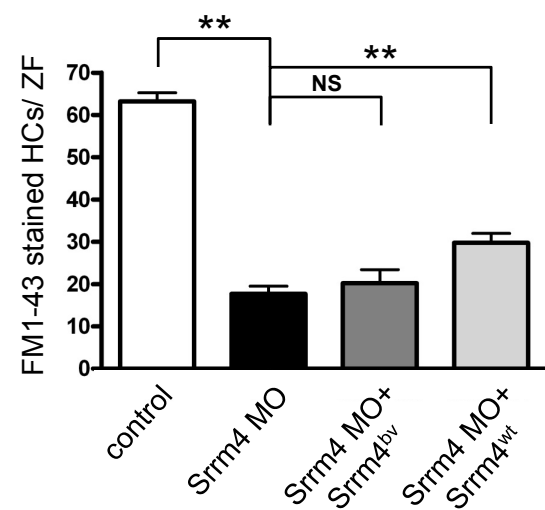

Supplement: Figure S10 — Functional analysis of Srrm4wt and Srrm4bv in HEK293 cells and zebrafish. (A) RT-PCR testing of alternative splicing in HEK293 cells transfected with both a minigene and a protein-encoding expression vector. The expression vectors encoded Srrm4wt, Srrm4bv, Srsf1, or no protein (vector control). Each minigene contained an alternative exon (red box), adjacent intronic sequences (∼300 bp each), and a constitutive exon at each end (white boxes). The promoter and polyadenylation site (pA) of the minigene cassette are indicated. The RT-PCR primers (arrows) were designed to anneal to the constitutive exons. Results obtained with 12 minigenes are shown. (B–C) Statistical analysis of the number of (B) mGFP-positive neuromasts and (C) FM1–43-stained hair cells (HCs) in the following groups of claudin B-mGFP transgenic zebrafish (ZF; 72 hpf): non-injected (control, n = 20), zSrrm4 MO-injected (n = 25), zSrrm4 MO- and zSrrm4bv-injected (n = 9), and Srrm4 MO- and zSrrm4wt-injected (n = 16) (one-way ANOVA, P<0.0001, post-hoc Bonferroni's test: *P<0.01, **P<0.001; NS: non-significant). (PDF) [file pgen.1002966.s010.pdf]
