# Supplementary material for: A Mutation in the Srrm4 Gene Causes Alternative Splicing Defects and Deafness in the Bronx Waltzer Mouse
Source: PLoS Genet. 2012 Oct 4;8(10):e1002966. doi: 10.1371/journal.pgen.1002966 (PMC3464207; doi:10.1371/journal.pgen.1002966)

Figure S11

A

```
Dtna pre-mRNA (mutated region)
WT: UGCUGCCAAAAG-UGAUACUUG-GUAAGUGAUGAAC
M:  UGCUGCCAAAAG-UGCCGGGUG-GUAAGUGAUGAAC
```

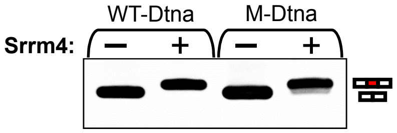

B

```
Dtna pre-mRNA (mutated region)
WT: UGCUGCCAAAAG-exon
M1: UACUGCCAAAAG-exon
M2: UGCUACCAAAAG-exon
M3: UACUACCAAAAG-exon
```

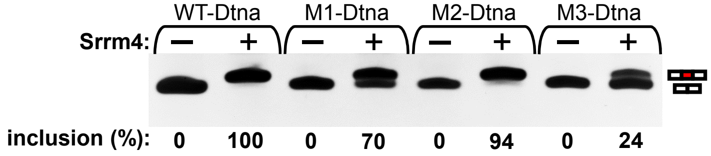


```
Mef2d pre-mRNA (mutated region)
WT: UGGUUUUAUUUCUGCUCCCAG-exon
M1: UAAUUUUAUUUCUGCUCCCAG-exon
M2: UGGUUUUAUUUCUACUCCCAG-exon
M3: UAAUUUUAUUUCUACUCCCAG-exon
```

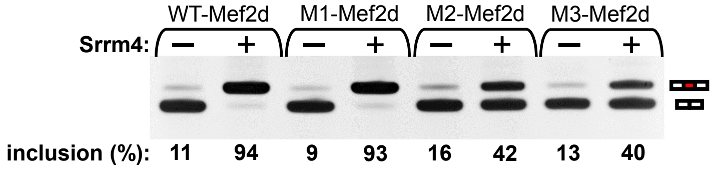


```
Sh3glb1 pre-mRNA (mutated region)
WT: UGCUGGCUAG-exon
M1: UACUGGCUAG-exon
M2: UGCUAACUAG-exon
M3: UACUAACUAG-exon
```

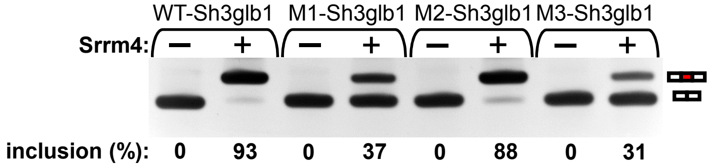


```
Kif1b pre-mRNA (mutated region)
WT: UGCUUUCGUUUGCAG-exon
M:  UACUUUCGUUUGCAG-exon
```

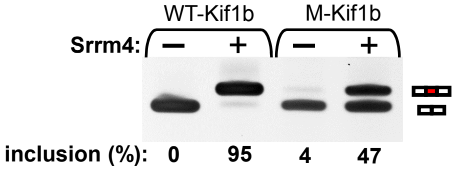

C

```
Vps13c pre-mRNA (mutated region)
WT: UGCUUCCACUGAGAAG-exon
M1: UACUUCCACUGAGAAG-exon
M2: UGUUUCCACUGAGAAG-exon
```

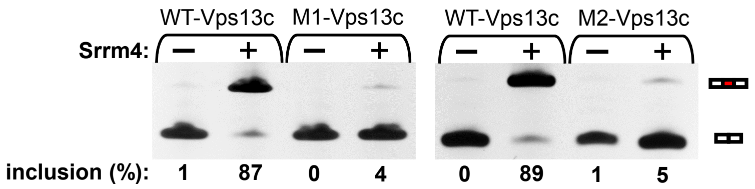

D

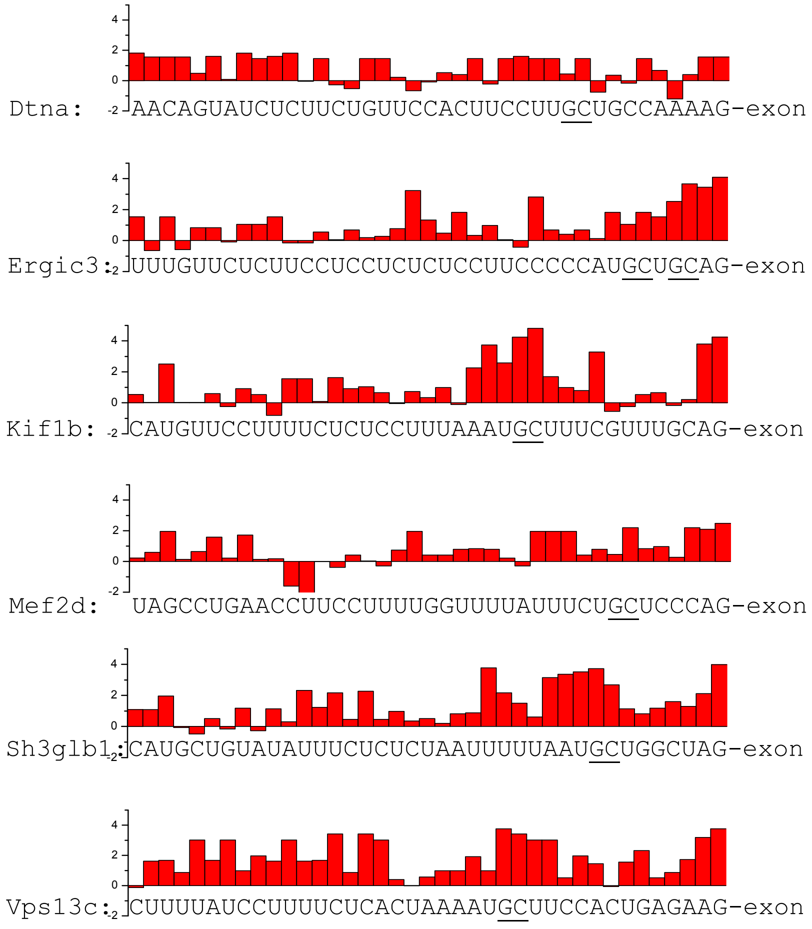

Supplement: Figure S11 — The effects of exon and intron mutations on the Srrm4-dependent inclusion of alternative exons into the mature mRNA. (A) RT-PCR testing of alternative splicing of control (WT) and mutated (M) Dtna exon 11. For the RT-PCR tests, RNA was extracted from HEK293 cells that were transfected with both a Srrm4 construct (Srrm4wt, +; or empty pcDNA3.1 vector, −) and a Dtna exon 11-containing minigene (control, WT; or mutant, M). The sequence of the alternative exon is highlighted by blue characters; the mutated nucleotides are indicated by bolding. The minigenes also contained the exon-flanking intron sequences (300–300 bp) from Dtna. (B–C) Effects of mutations in selected UGC sequences on Srrm4-dependent splicing. RT-PCR testing of alternative splicing in HEK293 cells transfected with both an Srrm4 construct (Srrm4wt, +; or empty pcDNA3.1 vector, −) and a minigene consisting of exons and introns. The control (WT) minigenes did not contain mutations, whereas the mutant minigenes (M, or M1–3) contained base substitutions. The relevant sequence fragments for each encoded pre-mRNA are shown. Bolding indicates the mutated bases; hyphens indicate the intron-exon borders; levels of exon inclusion are shown in percentages. G-to-A (B,C) and C-to-U (C) substitutions in the encoded pre-mRNAs were designed to alter selected GC motifs. (D) Nucleotide conservation in the intronic regions located immediately upstream of Srrm4-regulated alternative exons. The heights of red columns indicate the vertebrate base-wise conservation scores calculated by PhyloP [67]. Positive numbers indicate conservation, negative values indicate accelerated evolution. The underlined GC motifs contain the G nucleotides that were found to be important for Srrm4-dependent splicing. (PDF) [file pgen.1002966.s011.pdf]
